# Swiss ethnoveterinary knowledge on medicinal plants – a within-country comparison of Italian speaking regions with north-western German speaking regions

**DOI:** 10.1186/s13002-016-0106-y

**Published:** 2017-01-03

**Authors:** Maria Mayer, Mirjam Zbinden, Christan R. Vogl, Silvia Ivemeyer, Beat Meier, Michele Amorena, Ariane Maeschli, Matthias Hamburger, Michael Walkenhorst

**Affiliations:** 1 Faculty of Bioscience and Agri-Food and Environment Technologies, University of Teramo, Teramo, Italy; 2 Pharmaceutical Biology, Department of Pharmaceutical Sciences, University of Basel, Basel, Switzerland; 3 Division of Organic Farming, Department of Sustainable Agricultural Systems, University of Natural Resources and Life Sciences (BOKU), Vienna, Austria; 4 Farm Animal Behaviour and Husbandry Section, University of Kassel, Kassel, Germany; 5 Unit of Phytopharmacy and Natural Product Research, Institute of Chemistry and Biotechnology, Zurich University of Applied Sciences, Wädenswil, Switzerland; 6 Department of Livestock Science, Research Institute of Organic Agriculture, Ackerstrasse 113, Postfach, CH-5070 Frick Switzerland

**Keywords:** Ethnoveterinary, Herbal remedies, Switzerland, Italian speaking regions (Ticino, Grisons), German speaking cantons (Lucerne, Solothurn, Bern, Basel), Italy, Farmers

## Abstract

**Background:**

Ethnoveterinary knowledge in Europe may play an important role as a basis for sustainable treatment options for livestock. Aims of our study were (a) to compare the ethnoveterinary practices of two culturally and sociodemographically different regions of Switzerland, (b) to compare results with earlier ethnoveterinary studies conducted in Switzerland and in adjacent Italian regions and, (c) to evaluate possible reasons for regional differences in European ethnoveterinary medicine.

**Methods:**

25 interviews were conducted in 2014 in all Italian speaking regions (ItR) of Switzerland, and 31 interviews were held in five north-western German speaking Cantons (GeC). Semi-structured questionnaires were used to collect detailed information regarding plant species, mode of preparation, dosage, route of administration, category of use, origin of knowledge, frequency of use, and satisfaction with outcomes of the treatments.

**Results:**

A total of 162 homemade remedies in ItR and 219 in GeC were reported, out of which 125 and 145, respectively, were reported to contain only one plant species (homemade single species herbal remedy report, HSHR). 44 ItR and 43 GeC plant species were reported to treat livestock, of which only a half were used in both regions. For each HSHR, we classified the treatment intention of all use reports (UR), leading to a total of 205 and 219 UR in ItR and GeC respectively. While cattle were the most often treated livestock species in both study regions, in ItR 40% of UR were administered to small ruminants. Main indications in both regions were gastrointestinal diseases and skin afflictions, but in ItR a high number of URs were reported as antiparasitics. URs were mainly handed down from the past generation, but in GeC the source of knowledge for 20% of URs were from courses. Regarding the used plant species, ItR showed a higher concordance with Swiss than Italian studies, but with some differences to all regions. A total of 22 (14 ItR; 8 GeC) plant species in this study have not been reported before in ethnoveterinary studies of Swiss and Italian alpine regions.

**Conclusions:**

ItR and GeC, show differences and similarities with respect to their own ethnoveterinary practices and earlier Swiss and Italian ethnoveterinary studies. Linguistic, geographical, as well as social and farm-structural conditions influence the regional ethnoveterinary knowledge. However, political borders seem to be more important than language or geographical barriers.

**Electronic supplementary material:**

The online version of this article (doi:10.1186/s13002-016-0106-y) contains supplementary material, which is available to authorized users.

## Background

### Ethnoveterinary research

Ethnoveterinary research, defined by McCorkle as the “systematic investigation and application of veterinary folk knowledge, theory and practise” [1], is of raising importance in Europe [2, 3] even though conducted mostly in developing countries [3]. Traditional knowledge of medicinal plant use in animals has been recorded from 12 out of 37 European Union and affiliated countries, with Italy, Spain and Turkey being the most intensively investigated ones [2].

Homemade herbal remedies, handed down over generations, may be a useful therapeutic alternative for treatment of livestock [4, 5]. In addition, dissemination of such knowledge may raise awareness of the potential of veterinary phytotherapy [6]. While in developing countries animal health care is often based on self-made preparations, particularly when access to western veterinary products is difficult or too expensive [7], European farmers may opt for such “phytotherapeutic products” in order to comply with EU directives for organic livestock treatment [8]. Moreover, a considerable amount of veterinary antimicrobials is sold in EU each year (4,802 tonnes of active ingredient in 2012) [9] and since the most of this amount is directed to treat livestock species, cross contamination of resistant strains of zoonosis pathogens is more than a possibility [4, 9, 10]. Thus ethnoveterinary medicine may play an important role also in Europe [2].

Switzerland is one of several states which arouse out of the dissolution of the Holy Roman Empire, but differs profoundly in its cultural origins from other European countries. While other states often originated from the consolidation of princely houses domains, the core of Switzerland was born as voluntary union of small communities in a loose federation [11]. The Swiss Confederation expanded over centuries from a central German speaking core to 26 cantons of today. Cantons are relatively autonomous, and the sometimes significant cultural differences are the result of distinctly differing historical paths followed by individual cantons, and are reflected by four linguistic regions and official languages (German, French, Italian and Romansch) [11, 12]. While the topography of Switzerland was largely responsible for the development of cultural and linguistic regions, the political system of a federal republic established in the 19^th^ century was the basis for a preservation of cultural and linguistic plurality [12].

A comparison of ethnoveterinary data from Spain, Italy and Albania with those from the Romanian region of Transylvania shows only few overlaps [13]. On the other hand, the compared regions are distant from each other and belong to different linguistic regions. But even within the same country and linguistic region significant regional differences have been reported, as e.g. for four territories of the Catalan linguistic area of Spain [14]. In contrast, the comparison of ethnoveterinary data even from two continents (central and southern Italy, Europe, with Tunisia, Africa) shows that about one third of the plant species described for Tunisia are also used in Italy for veterinary purposes [15]. However, a comparative study of ethnoveterinary knowledge of two maximally different regions (regarding history, geography, farm size, farm structure and spoken language) within the same country, and with ethnoveterinary data from the same linguistic region in an neighbouring country has not been conducted up to now in central Europe. Switzerland seems to be predestined to address this question in an exemplary manner due to its large cultural, agricultural, geographical and linguistic diversity on the one hand, and the long history of territorial and political cohesion on the other.

### Study area

**Fig. 1:**
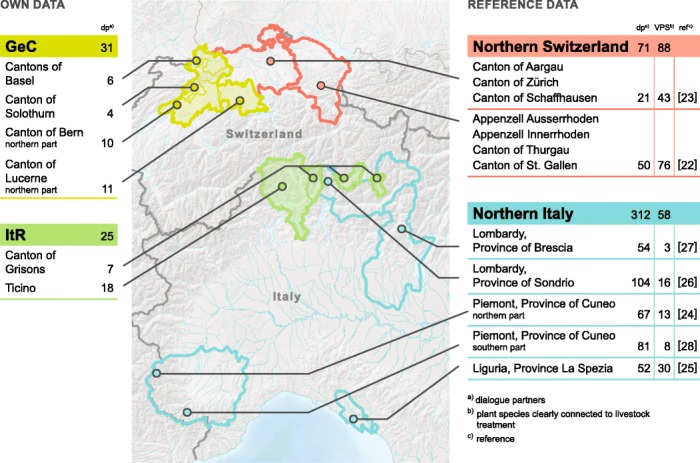
Map of Switzerland and northern Italy showing regions of own research (Italian speaking region (ItR) and German speaking cantons (GeC)) as well as research regions of reference data (Switzerland [22, 23] and northern Italy [24–28]) with the respective numbers of plant species and dialogue partners

The study area included two different regions of Switzerland: on the one hand, a climatically mild, low altitude area extending over five German speaking cantons (GeC) of central and north-western Switzerland (Solothurn, Basel Landschaft, Basel Stadt, the northern part of Lucerne, and the north-eastern German speaking part of Berne), and a southern, mainly mountainous area comprising the canton of Ticino and adjacent Italian speaking regions (ItR) in the canton of Grisons (Moesa district, Bernina district and Bregaglia municipality) (Fig. 1). Three major biogeographic regions can be distinguished in Switzerland, (i) the Jura, a low mountain range, (ii) the Central Plateau, an alluvial basin and, (iii) the Alps, a high mountain region. GeC represents mainly the Central Plateau, while ItR is part of the Alps. The Alps, in particular, are an area of high plant diversity.

Historically, GeC includes territories of the Old Federation, an association of confederates signing pacts of eternal alliance as early as 1332 (Lucerne), 1353 (Berne), 1481 (Solothurn) and 1501 (Basel), respectively [11]. The canton of Grisons became loosely associated with the Federation in 1498 [11], while the territories of Ticino belonged to the dukes of Milan until 1512, when they were conquered and by this way fell under “foreign” administration of the Federates. Grisons and Ticino became formally part of the Swiss Confederation in 1803 [12].

The area of the GeC is located between 46°96′–47°59′ North and 7°02′–8°51′ East in the north-western part of Switzerland, while ItR is located between 45°81′–46°63′ North and 8°38′–10°16′ East on the southern side of the Swiss Alps. The altitude of GeC is between 246 m and 1,592 m above sea level, with an average annual temperature of 9 °C and annual average precipitation of 1009 mm [16]. The altitude of the ItR is between 194 m and 3,402 m above sea level. Climate in ItR differs widely depending on altitude, with an average annual temperature in between 11,7° and −6 °C [16]. The average annual precipitation ranges between 789 and 2262 mm [16]. Both regions cover approximately 3,900 km^2^. ItR has about 360,000, GeC about 1,090,000 inhabitants [17].

In ItR, out of a total of 790 farms in Ticino, 122 are registered as organic (16%), while the canton of Grisons has the highest percentage of organic farms (55%; 1280 organic farms out of a total of 2298) in Switzerland. About 290 of all farms of Grisons are located in ItR. Due to the mountainous topography of the area, most often reared livestock species are cattle and small ruminants. Also due to missing arable land as base for own crop-feed production poultry is most commonly kept in small herds for self-supply, while pigs have almost no importance [17, 18].

GeC include more densely populated areas, and farms are generally larger and have significantly more cropland. Out of a total of 28,441 farms in the 4 cantons, the area where interviews were carried out counts approximately 10,000 farms. In Bern 212 (5%) of 4,046, in Luzern 234 (7%) of 3,507, in Solothurn 124 (8%) of 1,508, and in both Basel cantons 128 (13%) of 978 were organic farms. The most often reared livestock species were cattle. Small ruminants are negligible, while poultry and pigs have a high importance in the rural economy [17, 18].

### Aim of the study

Ethnobotany, the “study of the relationship between human beings and vegetation in their environment” [19] is “an interdisciplinary approach, including anthropology, archaeology, botany, chemistry, ecology, pharmacology and psychology (that) leads us to understand the relationship between plants and human societies” [20]. Ethnoveterinary studies have shown differences and similarities between and even within countries [14, 21]. However, a comparison of two maximally different areas within the same country has not been conducted so far. Regarding history, spoken language, geography, farm size and farm structure ItR and GeC represent probably the highest difference within Switzerland. Due to the fact that ItR and Italy belong to the same linguistic area we expected here a close similarity.

The aims of our study were (a) to compare the ethnoveterinary practices of two cultural and sociodemographic different regions of Switzerland, ItR and GeC, (b) to analyse to what extent the ethnoveterinary practices in both regions were in accord with earlier studies conducted in neighbouring areas of Switzerland [22, 23] and Italy [24–28] and, (c) to estimate if and how far political borders, language and other reasons could be responsible for regional differences in European ethnoveterinary medicine.

## Methods

### Dialogue partners

Several strategies were used to identify dialogue partners, as described in previous studies [22, 23]. As a first step, a detailed letter with information about the project and its aim was sent to all organic farmers in the study area with help from local organizations of organic producers (“Bärner Bio Bure”, “Bio Luzern”, “Bio Nordwestschweiz”, “Bio Grischun” and “Bio Ticino”). In addition, the project was personally presented at two farmer meetings, both in ItR and GeC. A broader population was informed through publications in the local agricultural press and on websites. In GeC a letter was also sent to all pig farmers, contacted via Swiss Swine Health Service, and some more contacts came from the dairy and poultry farm research network of the Research Institute of Organic Agriculture (FiBL, Frick, CH). Persons contacted were asked to support the project, either as dialogue partners (persons who offers their own ethnoveterinary knowledge regarding herbal remedies to the project; all persons who offered their ethnoveterinary knowledge were interviewed), or as informants providing information on other farmers, leading to further dialogue partners [29].

In ItR, interviews were carried out from end of February to mid of August 2014, with a total of 25 dialogue partners. In GeC, a total of 31 dialogue partners were interviewed from the beginning of March to the end of April 2014. In most of the cases dialogue partners were interviewed alone. However, in six farms in ItR, and in nine cases in GeC further family members assisted during the interview. Information therefore came from 31 different persons in ItR, and 41 in GeC. Nevertheless, information provided by family members of farmers were added to the data of the main dialogue partner and not analysed separately [22]. Interviewed persons were all active or retired farmers. In ItR a total of 15 women and 16 men took part in the interviews, in GeC 21 women and 20 men. The age of the dialogue partners or assisting persons was between 30 and 80 (mean 58 ± 13) years in ItR, and between 33 and 77 (mean 56 ± 11) years in GeC, respectively. The distribution of interviews between the Cantons was as follows: 10 were held in Bern, 4 in Solothurn, 11 in Luzern, 6 in Basel (GeC); 18 in Ticino and 7 in Grisons (ItR) (Fig. 1).

### Farms

In ItR, the altitude of farms varied considerably, as the 25 farms were located between 300 m and 1800 m above sea level. Five farms were below 500 m, 7 were between 500 m and 899 m, 7 between 900 and 1100 m, and the 6 remaining farms on an altitude above 1100 m. The ItR sample included 15 (60%) organic and 10 (40%) non-organic farms. Nineteen out of 25 (76%) interviewed ItR farms kept cattle, 12 (48%) goats, and 9 (36%) sheep. In addition, 15 (60%) kept a small number of lying hens or meat broiler, 10 (40%) pigs, 4 (16%) bees, 3 (12%) rabbits, and one (4%) ducks with almost all the production being for consumption by the family. Six farms (24%) kept horses as companion animals, and 6 (24%) asses or mules as working animals.

The location of the 31 farms in GeC varied between 460 m and 950 m above sea level. Four farms were below 500 m, 26 were between 500 m and 899 m, and one farm was between 900 and 1100 m. Sixteen (52%) farms were organic, and 15 (48%) non-organic. Twenty-three out of the 31 farms (74%) kept cattle, 16 (52%) poultry and 13 (42%) pigs.

### Interview process

Interviews were conducted following the same structure of previous studies [22, 23]. Before starting the interview, the dialogue partners were asked to give a written agreement for recording the interview (by OLYMPUS WS 200S Digital Voice Recorder, Olympus Imaging Europa GmbH, Hamburg, Germany). Recorded interviews were not transcribed, but used for cross checks in case of inconsistencies or missing data in the questionnaires. An interview usually took between one and three hours. Final data were entered into a database [30].

Allmost all interviews were conducted in winter and early spring, so that no voucher specimens could be taken from the plants mentioned by the dialogue partners. Plants were identified via their local vernacular names. Illustrations of the Flora Helvetica [31] were used for confirmation by the dialogue partner. Based on the Flora Helvetica [31] the availability of the plant species in the study area was cross checked. In case of uncertainties names and, if available, plant material available at the farms, were cross checked for plausibility with information collected during earlier studies [22]. In case of purchased plant material the package or the package leaflet was used to identify the plant species.

In the context of some few interviews conducted in the summer a total of 7 herbarium voucher specimen of 7 plant species gathered in the wild were collected together with the dialogue partner, dried, labelled, and deposited at the herbarium of the “Basler Botanische Gesellschaft”, Botanical Institute of the University of Basel (Schönbeinstrasse 6, 4056 Basel, Switzerland). All species of which voucher specimens were available from our own or from former Swiss studies [22] where marked in Additional file 1.

During each interview, detailed information about the used plants, the manufacturing process, the use report, as well as the administration of homemade remedies were gathered. To evaluate concentration in g of dry plant in 100 g of finished product and oral daily doses the amount of plant used was determined on site using a precision scale (Kern PCB, Kern & Sohn GmbH, Germany). This operation was carried out with the original plant parts of the farmer, or with reference drugs provided by the interviewer, namely a collection of herbal drugs of Pharmacopoeia quality [32]. If this was not possible, dosages were estimated by measurement of the administered volume of the mentioned plant and weighting of the material afterwards.

Interview partners were asked to indicate the use as free answer. The information was afterwards classified following the anatomical therapeutic chemical classification system for veterinary medicinal products ATCvet [33]. Routes of administration, as well as the daily frequency and mean duration of treatment were also recorded. The routes of administration were classified as internal (oral or intravaginal/intrauterine) and external administration (on intact or on altered/sore skin), following earlier studies [22, 23].

The daily dosage was calculated for orally administered medicinal plant (g of dry plant equivalent) and was then normalized into dosage per kilogram metabolic body weight(MBW = bodyweight^0.75^) to allow a comparison between different species (including human) [22, 23, 34, 35]. The following formula was used:

$$ \mathrm{Daily}\ \mathrm{dose}\ \left(\mathrm{g}/\mathrm{k}{\mathrm{g}}^{0.75}\right)=\frac{drug\  dose\  per\  administration\ (g)\ x\  repetition\  per\  day}{metabolic\  bodyweight\ \left(k{g}^{0.75}\right)} $$

In case of topical use, the concentration of herbal preparations in g dry plant equivalent per 100 g of finished product was calculated.

Further information about frequency of use, origin of knowledge, and optional use of additional therapies was collected by the interviewer. To evaluate the satisfaction regarding the outcome of each use report a 100 mm visual analogue scale (VAS) was utilized, with 0 mm corresponding with “no effect”, and 100 to “very good effect”. Means of the VAS were calculated for each study region.

### Definitions

We used the following definitions [22]:

#### Homemade remedy report

We define homemade remedy report as follows: [dialogue partner] x [plant species or other natural compounds] x [plant part] x [manufacturing process to the finished product].

#### Use report

We define use report (UR) as follows: [homemade remedy report] x [category of use] x [specification of use] x [animal species] x [animal age classification] x [administration procedure].

## Results

During the interviews in ItR 25 dialogue partners reported between 1 and 16 (mean 6 ± 4 each), and a total of 162 homemade remedy reports. Forty-nine different plant species belonging to 30 plant families were mentioned. Out of the 162 homemade remedy reports, 125 (77%) were prepared with only one plant species (homemade single-species herbal remedy reports, HSHR), 9 (6%) mixtures contained two to four plants (one mixture included in addition one lichen), and 28 (17%) homemade remedy reports did not involve plants (HRWP), but, e.g. lard, albumen, vinegar, salt, sugar, quartz stone, phosphorus, and grappa.

In GeC, a total of 219 homemade remedy reports could be documented from 31 dialogue partners. Per interview between one and 14 homemade remedies (mean 7 ± 3) were described, corresponding to 57 plant species of 34 botanical families. In total 145 out of the 219 homemade remedies (66%) were classified as HSHR, 18 (8%) mixtures contained two to seven plant species, and 56 (26%) HRWP could be documented e.g. vinegar, schnapps, honey, albumen, chicken manure, and tar.

### Composition and manufacturing process of ItR-HSHR and GeC-HSHR

Out of the 125 ItR-HSHR, plants belonging to the Asteraceae family were the most frequently mentioned, with a total of 33 HSHR (26%), followed by Linaceae and Urticaceae (13 ItR-HSHR each, 10%). *Calendula officinalis* L. (15 ItR-HSHR, 12%), *Linum usitatissimum* L.(13 ItR-HSHR, 10%) and Coffea spp. (9 ItR-HSHR, 7%) were the species with the highest number of reports, while *Arnica montana* L., *Urtica urens* L. and *Malva neglecta* Wallr. were each single ingredient of 7 ItR-HSHR (6%) (Table 1).

**Table 1:** Extraction procedure to prepare homemade single-species herbal remedy reports (HSHR): a: 125 HSHR from Italian speaking regions (ItR-HSHR) named by 25 farmers

Botanical family (Number of named plant species in this family)	Plant species with ≥ 3 named IsR‐HSHR (Numbers indicate the frequency of mentioned 125 ItR‐HSHR)	On farm extraction procedure									
			None	Water			Alcohol		Oil/Fat		Honey
		CP		Room temperature	Infusion /Decoction		Room temperature	Heated up	Room temperature	Heated up	
Asteraceae (7)	all Asteraceae (33)	7	1	1	6	2	9		2	5	
	*** Calendula officinalis*** **L.** (15)										
	Flos and flos sine calice (14)	6^a^					2		2	4	
	Herba (1)									1	
	*** Arnica montana*** **L.** (7)										
	Flos (6)			1			5				
	Herba cum radice (1)						1				
	*** Matricaria recutita*** **L**. (5)										
	Flos (5)				4	1					
	other Asteraceae^1^ (6) **[vs]**	1^b^	1		2	1	1				
Linaceae (1)	*** Linum usitatissimum*** **L.** (13)										
	Semen (13)		3	8		2^c^					
Urticaceae (2)	all Urticaceae *(13)*		11		1	1					
	*** Urtica urens L.*** *(7)*										
	Herba (7)		5		1	1					
	*** Urtica dioica*** **L.** (6) **[vs]**										
	Herba (6)		6								
Malvaceae (2)	all Malvaceae (10)				6	4					
	*** Malva neglecta*** **Wallr.** (7)										
	Folium (3)				2	1					
	Herba (2)				2						
	Herba cum radice (2)					2					
	*** Malva sylvestris L.*** (3)										
	Folium (1)				1						
	Herba (2)				1	1					
Rubiaceae (1)	** Coffea spp** (9)										
	Semen (9)					9					
Poaceae (4)	all Poaceae (6)		2	1		3					
	*** Oryza sativa*** **L.** (3)										
	Semen					3					
	other Poaceae^2^ (3)		2	1							
Liliaceae (2)	all Liliaceae (5)		4								1
	*** Allium sativum L.*** (4)										
	Bulbus (4)		4								
	other Liliaceae^3^ (1)										1
Hypericaceae (1)	*** Hypericum perforatum*** **L. (4) [vs]**										
	Flos (3)								3		
	Herba (1)								1		
Pinaceae (2)	all Pinaceae (4)		4								
	*** Picea abies*** **(L.) H. Karst**. (3)										
	Resina (3)		3								
	other Pinaceae^4^ (1)		1								
**others (22)**	**other plant species** ^**5**^ **(28) [vs]**	1^d^	18		5	2		2			
**Total (44)**		**8**	**43**	**10**	**18**	**23**	**9**	**2**	**6**	**5**	**1**

[vs]: voucher specimens are accessible via species name, year of sampling (2014), and name of the first author (Maria Mayer), at the herbarium of the “Basler Botanische Gesellschaft”, Botanical Institute of the University of Basel (Schönbeinstrasse 6, 4056 Basel, Switzerland)

^1^Asteraceae (4): *Alchemilla millefolium* L. (2) [vs], *Artemisia absinthium* L. (1) [vs], *Artemisia campestris* L. (1), *Taraxacum officinale* WEB. Ex Wigg. (2); ^2^ Poaceae (3): *Avena sativa* L. (1); *Triticum aestivum* L. (1); *Hordeum vulgare* L. S.L. (1); ^3^ Liliaceae (1): *Allium cepa* L. (1); ^4^ Pinaceae (1): *Abies alba* Mill. (1); ^5^ Amaryllidaceae (1): *Allium orsinum* L. (1); Apiaceae (1): *Foeniculum vulgare* Mill. (2); Aspidaceae (1): *Dryopteris filix-mas* L. (SCHOTT) (1) [vs]; Boraginaceae (1): *Symphytum officinale* L. (1); Brassicaceae (1): *Brassica oleracea* L. (convar. Capitata var. Sabauda L.) (1); Caryophyllaceae (1): *Stellaria media* (L.) VILL. (2) [vs]; Cucurbitaceae (1): *Cucurbita maxima* Duch. (1); Euphorbiaceae (1): *Ricinus communis* L. (1); Fagaceae (2): *Castanea sativa* Mill. (1); *Quercus robur* L. (1); Gentianaceae (1): *Gentiana purpurea* L. (2); Juglandaceae (1): *Juglans regia* L. (1); Lamiaceae (2): *Lavandula angustifolia* Mill. (1); *Salvia verbenacea* L. (1); Lauraceae (1): *Cinnamomum verum* J.Presl. (1); Loranthaceae (1): *Viscum album* L. S.L. (1); Myrtaceae (1): *Eugenia caryophyllata* Thunb. (1); Oleaceae (1): *Olea europaea* L. (1); Rhamnaceae (1): *Rhamnus catharticus* L. (2) [vs]; Rosaceae (1): *Potentilla erecta* (L.) RÄUSCHEL (1); Scrophilariaceae (1): *Euphrasia rostkoviana* Hayne, 1985 (2); Solanaceae (1): *Nicotiana tabacum* L. (1);

CP: commercial products

^a^ *Calendula* tincture (pharmacy) used in four remedies; *Calendula* ointment by Weleda® (pharmacy) used in two remedies; ^b^ Dandelion tincture (pharmacy) used in one remedy; ^c^ In two remedies, linseeds were boiled in the same water were they were kept for a whole night; d Castor oil (*Ricinus communis* seeds oil) used in one remedy

Considering the 145 GeC-HSHR, the Asteraceae family was represented in 36 (25%) GeC-HSHR. Rubiaceae, Boraginaceae families were each mentioned in 9 (6%) GeC-HSHR, while Urticaceae and Rhamnaceae were mentioned in 8 (6%) GeC-HSHR. Eighteen (12%) GeC-HSHR contained *Matricaria recutita* L., while Coffea spp L. and *Symphytum officinale* L. represented 9 GeC-HSHR (6%) each. *Calendula officinalis* L*.*, *Urtica dioica* L*.* and *Rhamnus catharticus* L. were contained in 8 GeC-HSHR (6%) each (Table 2).

**Table 2:** Extraction procedure to prepare homemade single-species herbal remedy reports (HSHR): 145 HSHR from German speaking Cantons (GeC‐HSHR) named by 31 farmers

Botanical family	Plant species witch ≥ 3 named GeC‐HSHR		On farm extraction procedure						
(Number of named plants species in this family)	(Number indicate the frequency of mentioned 145 GeC‐HSHR)	CP	None	Water			Alcohol	Oil/Fat	
				Room temperature	Infusion	Decoction	Room temperature	Room temperature	Heated up
Asteraceae *(5)*	all Asteraceae (36)	*5*	*1*		*13*	*7*	*3*	*3*	*4*
	*** Matricaria recutita*** ** L. (18)**								
	Flos (15)	1			8	5	1		
	Herba (3)				2	1			
	*** Calendula officinalis*** **L. (8)**								
	Flos (8)	1						3	4
	*** Arnica montana*** **L. (5)**								
	Flos (5)	3					2		
	*** Achillea millefolium*** **L. agg. (4)**								
	Herba (3)		1		2				
	Flos (1)					1			
	other Asteraceae^1^ (1)				1				
Rubiaceae (1)	** Coffea spp (9)**								
	Semen (9)				9				
Boraginaceae (1)	*** Symphytum officinale*** **L. agg. (9)**								
	Radix (8)	4				1	1		2
	Folium (1)		1						
Urticaceae (1)	*** Urtica dioica*** **L. agg. (8)**								
	Herba (4)		4						
	Folium (4)		3		1				
Fagaceae (1)	*** Quercus robur*** **L. agg. (7)**								
	Cortex (7)		4		2	1			
Lamiaceae (2)	all Lamiaceae (7)	1	3		3				
	*** Thymus vulgaris*** **L. (6)**								
	Herba (6)		3		3				
	other Lamiaceae^2^ (1)	1							
Rosaceae (4)	all Rosaceae (7)		2	2	1	1	1		
	***Potentilla erecta*** **(L.) Raeusch agg. (3)**.								
	Rhizoma (3)			2 ^a^			1		
	other Rosaceae^3^ (4)		2		1	1			
Rhamnaceae (1)	*** Rhamnus cathartica*** **L. (8)**								
	Herba (8)		8						
Polygonaceae (1)	*** Rumex obtusifolius*** **L. agg. (6)**								
	Radix (3)					3			
	Folium (2)						1	1	
	Herba cum radice (1)		1						
Theaceae (1)	***Camellia sinensis*** **(L.) O. Kuntze. (5)**								
	Folium (5)				5				
Brassicaceae (2)	all Brassicaceae (5)		4		1				
	***Armoracia rusticana*** **P. Gaertn. & al. (4)**								
	Radix (4)		3		1				
	other Brassicaceae^4^ (1)		1						
Hypericaceae (1)	*** Hypericum perforatum*** **L. agg. (3)**								
	Flos (2)							2	
	Herba (1)							1	
Linaceae (1)	*** Linum usitatissimum*** **L. agg. (3)**								
	Semen (3)		1			2			
Berberidaceae (1)	*** Berberis vulgaris*** **L. (3)**								
	Herba (3)		3						
Others (20)	other plant species ^5^ (29)	4	8	2	4	6		2	3
Total (43)		14	43	4	39	21	6	9	9

^1^Asteraceae (1): *Artemisia absinthium* L. (1); ^2^Lamiaceae (1): *Origanum vulgare* L. agg. (1); ^3^ Rosaceae (3): *Alchemilla mollis* (Buser) Rothm. (2), *Malus domestica* Borkh. (1), *Potentilla anserina* L. (1); ^4^Brassicaceae (1): *Capsella bursa‐pastoris* (L.) Medikus agg. (1); ^5^ Pinaceae (2): *Abies alba* Mill. (2), *Picea abies* (L.) H. Karst. (2), Malvaceae (2): *Althaea officinalis* L. (2), *Malva sylvestris* L. (1), Apiaceae (2): *Foeniculum vulgare* Mill. (2), *Carum carvi* L. (1), Cannabaceae (1): *Cannabis sativa* L. (2), Aspidiaceae (1): *Dryopteris filix‐mas* L. (2), Araliaceae (1): *Panax ginseng* C.A. Meyer (2), Lauraceae (2): *Laurus nobilis* (1), *Cinnamomum camphora* L. (1), Poaceae (1): *Avena sativa* L. (2), Scrophulariaceae (1): *Euphrasia officinalis* L. (2), Juglandaceae (1): *Juglans regia* L. (1), Ericaceae (1): *Arctostaphylos uva‐ursi* (L.) Sprengel (1), Caprifoliaceae (1): *Sambucus nigra* L. (1), Xanthorrhoeaceae (1): *Aloe vera* (L.) Burm.f. (1), Papaveraceae (1): *Chelidonium majus* L. (1), Rutaceae (1): *Citrus* x *limon* (L.) Burm.f. (1), Liliaceae (1): *Allium cepa* L. (1) ^a^ Rosaceae; *Potentilla erecta* (L.) Raeusch. agg.: 1x extraction with milk

CP: commercial products

Aqueous extraction was the main processing of plants in both regions (51 ItR-HSHR, 41%; 64 GeC-HSHR, 44%). Direct use of plant parts without an extraction process came second (43 ItR-HSHR, 34%; 43 GeC-HSHR, 30%). In both regions also alcohol or oil/fat was used as extracting agent, and the use of some commercial products based on plants were reported (Tables 1 and 2). Farmers in ItR prepared 6 ointments, while in GeC 15 ointments were recorded. In 4 ItR-HSHR and 6 GeC-HSHR lard served as both extraction agent and ointment base. In 2 ItR-HSHR and in 7 GeC-HSHR beeswax was used as additional ointment base. In 2 GeC-HSHR milking grease (mineral ointment) alone or in combination with beeswax was the ointment-base.

Concentration of dry plant equivalent in the end-product was estimated for 95 ItR-HSHR (76%), either by using plant material provided by the interview partner (17 ItR-HSHR, 13.5%), with plant samples of our collection of herbal drugs (37 ItR-HSHR, 29.5%), or by assessment of administered volume of mentioned plant and subsequent weighing (41 ItR-HSHR, 33%). For 30 ItR-HSHR (24%), no estimation was possible. The same procedure was used in GeC, leading to estimation of concentration of plant equivalent in the final product for 84 GeC-HSHR (58%): in 33 GeC-HSHR (23%) by weighting the original plant, in 40 GeC-HSHR (27.5%) with our reference drugs, and in 11 cases (7.5%) the concentration was estimated by assessment of administered volume of mentioned plant and subsequent weighing. For further 61 GeC-HSHR (42%) it was not possible to estimate plant weight.

### Categories of use and routes of administration of ItR-use reports (ItR-UR) and GeC-UR

**Fig. 2:**
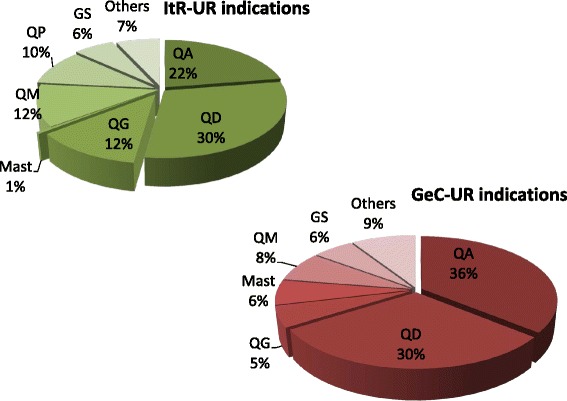
Distribution of use reports (UR) in Italian speaking region (ItR) and German speaking cantons (GeC). URs are grouped according to indications of the Anatomical Therapeutic Chemical Classification system for veterinary medicinal products (ATCvet). (QA = Alimentary tract and metabolism; QD = Dermatologicals; QG = Genito urinary system and sex hormones; Mast = Mastitis; QM = Musculo-skeletal system; QP = Antiparasitic products, insecticides and repellents; GS = General strengthening; “Others” for ItR-UR: QR (respiratory tract) agents, QS (sensory organs) agents, QN (nervous system) agent, various; “Others” for GeC-UR = QP, QR and various; Percentages referred to a total of 205 ItR-UR and 209 GeC-UR)

A complete list of ItR-UR and GeC-UR is available in Additional file 1. A total of 205 ItR-UR were reported for the 125 ItR-HSHR (Table 3). Thirty-nine of these (19%) were used as prophylactics, while 166 ItR-UR (81%) were therapeutics. For the 145 GeC-HSHR a total of 209 GeC-UR were described (Table 4), of which 199 GeC-UR (95%) were used for therapy, and 10 as prophylactics (5%). In both regions topical and oral administration were most frequently employed, and afflictions of the skin and the gastrointestinal tract were the main categories of use (Tables 3 and 4, Fig. 2). Ruminants were predominant as target animal species in both research regions. In ItR there was a nearly equal distribution between small ruminants and cattle, while in GeC mainly cattle were the intention of the treatment.

**Table 3:** Use Reports (UR) ‐ Information on routes of administration, categories of use, and target animal species are listed: 205 UR from Italian speaking regions (ItR‐UR), based on 125 homemade herbal remedy reports containing a single herb

Botanical family (Number of named plant species in this family)	Plant species with ≥ 3 named ItR‐HSHR (Number of named remedy reports are given in brackets)	(Numbers indicate the frequency of mentioned use reports)																		
		Routes of administration					Categories of use								Target animal species					Total different use reports
		External		Internal		Treatment of housing environment														
		I	A	O	U		QA	QD	QG	Mast	QM	QP	GS	Others^6^	C	S	G	Ns	Others^7^	
Asteraceae (7)	all Asteraceae (33)	19	36	18		1	8	40	4		19	1		2	23	20	18		13	74
	*** Calendula officinalis*** **L. (15)**																			
	Flos and flos sine calice (14)	7	23					24			6				9	8	8		5	30
	Herba (1)		3					3							1	2				3
	*** Arnica montana*** **L. (7)**																			
	Flos (6)	7	7					7			7				4	5	4		1	14
	Herba cum radice (1)	4		3					1		6				2				3	7
	*** Matricaria recutita*** **L. (5)**																			
	Flos (5)	1	3	7			5	4						2	6	2			1	11
	other Asteraceae^1^ *(6)*			8		1	3	2	3			1			1	3	2		3	9
Linaceae (1)	*** Linum usitatissimum*** **L. (13)**																			
	Semen (13)			15	1		2		9					5	8	1	4		3	16
Urticaceae (2)	all Urticaceae (13)	2		23			9		2				13	1	5		4		16	25
	*** Urtica urens*** L. (7)																			
	Herba (7)	1		14			9		1				5		4		3		8	15
	*** Urtica dioica*** **L. (6)**																			
	Herba (6)	1		9					1				8	1	1		1		8	10
Malvaceae (2)	all Malvaceae (10)	1	10	5	1		4	12	1						12		4		1	17
	*** Malva neglecta*** **Wallr. (7)**																			
	Folium (3)	1		2	1		2	1	1						3		1			4
	Herba (2)		2	1				3							1		2			3
	Herba cum radice (2)		5					5							5					5
	*** Malva sylvestris*** **L. (3)**																			
	Folium (1)			2			2								1		1			2
	Herba (2)		3					3							2				1	3
Rubiaceae (1)	** Coffea spp. (9)**																			
	Semen (9)			11			7		4						5	2	4			11
Poaceae (4)	all Poaceae (6)			7			4			3					4		3			7
	*** Oryza sativa*** **L. (3)**																			
	Semen			4			4								3		1			4
	other Poaceae^2^ (3)			3					3						1		2			3
Liliaceae (2)	all Liliaceae (5)			10								9		1	2		1		7	10
	*** Allium sativum*** **L. (4)**																			
	Bulbus (4)			9								9			2		1		6	9
	other Liliaceae^3^ (1)			1										1					1	1
Hypericaceae (1)	*** Hypericum perforatum*** **L. (4)**																			
	Flos (3)	2	2					2		1	1					3			1	4
	Herba (1)		1	1				1						1			1	1		2
Pinaceae (2)	all Pinaceae (4)	1	3	2				1			3	2				2	3	1		6
	*** Picea abies*** **(L.) H. Karst. (3)**																			
	Resina (3)	1	3					1			3					2	1	1		4
	other Pinaceae^4^ (1)			2								2					2			2
others (22)	other plant species^5^ (28)	4	5	20		4	12	5	2		1	9		4	11	4	9	2	7	33
Total (44)		29	57	112	2	5	46	61	25	1	24	21	13	14	70	32	51	4	48	205

**I** – intact skin; **A** – altered or sore skin; **O** – oral; **U** – intravaginal/intrauterine; **QA** – gastrointestinal disorders and metabolic dysfunctions; **QD** – skin afflictions and sores; **QG** – genito-urinary system and sex hormones (including peri-partum preparation); **Mast** – mastitis; Q**M** – musculoskeletal system (including hematomas and edema in the connective tissue); **QP** – antiparasitic products, insecticides and repellents; **GS** – general strengthening; **C** – Cattle; **S** – Sheep; **G** – Goat; **Ns** – No specification of the animal species (external administration);

^1^ Asteraceae (4): *Achilea millefolium* L. (2), *Artemisia absinthium* L. (1), *Artemisia campestris* L. (1), *Taraxacum officinale* WEB. Ex Wigg. (2); ^2^ Poaceae (3): *Avena sativa* L. (1); *Triticum aestivum* L. (1); *Hordeum vulgare* L. S.L. (1); ^3^ Liliaceae (1): *Allium cepa* L. (1); ^4^ Pinaceae (1): *Abies alba* Mill. (1); ^5^ Amaryllidaceae (1): *Allium ursinum* L. (1); Apiaceae (1): *Foeniculum vulgare* Mill. (2); Aspidaceae (1): *Dryopteris filix‐mas* L. (SCHOTT) (1); Boraginaceae (1): *Symphytum officinale* L. (1); Brassicaceae (1): *Brassica oleracea* L. (convar. Capitata var. Sabauda L.) (1); Caryophyllaceae (1): *Stellaria media* (L.) VILL. (2); Cucurbitaceae (1): *Cucurbita maxima* Duch. (1); Euphorbiaceae (1): *Ricinus communis* L. (1); Fagaceae (2): *Castanea sativa* Mill. (1); *Quercus robur* L. (1); Gentianaceae (1): *Gentiana purpurea* L. (2); Juglandaceae (1): *Juglans regia* L. (1); Lamiaceae (2): *Lavandula angustifolia* Mill. (1); *Salvia verbenacea* L. (1); Lauraceae (1): *Cinnamomum verum* J.Presl. (1); Loranthaceae (1): *Viscum album* L. S.L. (1); Myrtaceae (1): *Eugenia caryophyllata* Thunb. (1); Oleaceae (1): *Olea europaea* L. (1); Rhamnaceae (1): *Rhamnus catharticus* L. (2); Rosaceae (1): *Potentilla erecta* (L.) RÄUSCHEL (1); Scrophulariaceae (1): *Euphrasia rostkoviana* Hayne, 1985 (2); Solanaceae (1): *Nicotiana tabacum* L. (1); ^6^ QR respiratory tract ; QS sensory organ ; QN nervous system; various uses; 7 horses, pigs, donkeys, dogs, cats, hens

**Table 4:** Use Reports (UR) ‐ Information on routes of administration, categories of use, and target animal species are listed: 209 UR from German speaking Cantons (GeC), based on 145 homemade herbal remedy reports containing a single herb

Botanical family (Number of named plants species in this family)	Plant species witch ≥ 3 named GeC‐HSHR (Number indicate the frequency of mentioned 145 GeC‐HSHR)																
		Route of administration				Category of use (ATCvet Code)							Target animal species				Total different use reports
		External		Internal	Treatment of housing environment												
		I	A	O		QA	QD	QG	QM	Mast	GS	Others^6^	C	G	P	Others ^7^	
Asteraceae (5)	all Asteraceae (36)	10	26	28		24	29	3	3	3	1	2	75	5	3	8	91
	*** Matricaria recutita*** **L. (17)**																
	Flos (14)	1	12	16		16	11	2				1	23		3	3	29
	Herba (3)			4		3					1		4				4
	*** Calendula officinalis*** **L. (7)**																
	Flos (7)	1	15				15			1			12	1		3	16
	*** Arnica montana*** **L. (5)**																
	Flos (5)	5	2	2			3	1	3	2			5	4			9
	*** Achillea millefolium*** **L. (4)**																
	Herba (3)			3		2						1	1			2	3
	Flos (1)			2		2							2				2
	other Asteraceae^1^ (1)			1		1							1				1
Rubiaceae (1)	** Coffea spp (9)**																
	Semen (9)			11		7					3	1	11				11
Boraginaceae (1)	*** Symphytum officinale*** **L. agg. (9)**																
	Radix (4)	11	4				4		8	3			9		6		15
	Folium (1)			2		2							2				2
Urticaceae (1)	*** Urtica dioica*** **L. agg. (8)**																
	Herba (4)			6		5					1		3	1	1	1	6
	Folium (4)			5		1						4	4		1		5
Fagaceae (1)	*** Quercus robur*** **L. agg. (7)**																
	Cortex (7)			7		7							5		2		7
Lamiaceae (2)	all Lamiaceae (7)	1		12		5	1			1		6	10		1	2	13
	*** Thymus vulgaris*** L. (6)																
	Herba (6)	1		10		3	1			1		6	10		1		11
	other Lamiaceae^2^ (1)		2			2											2
Rosaceae (4)	all Rosaceae (7)		5	5		3	5	2					9		1		10
	*** Potentilla erecta*** **(L.) Raeusch. agg. (3)**																
	Rhizoma (3)		3	2		2	3						4		1		5
	other Rosaceae^3^ (4)		2	3		1	2	2					5				5
Rhamnaceae (1)	*** Rhamnus cathartica*** **L. (8)**																
	Herba (8)				8		8						8				8
Polygonaceae (1)	*** Rumex obtusifolius*** L. agg. (6)																
	Radix (3)			3		3							3				3
	Folium (2)	1	4	1			4		2				6				6
	Herba cum radice (1)			1		1							1				1
Theaceae (1)	***Camellia sinensis*** **(L.) O. Kuntze. (5)**																
	Folium (5)			5		5							5				5
Brassicaceae (2)	all Brassicaceae (5)	2		4		2		1	1	1	1		5		1		6
	*** Armoracia rusticana*** **P. Gaertn. & al. (4)**																
	Radix (4)	2		3		2			1	1	1		4		1		5
	other Brassicaceae^4^ (1)			1				1					1				1
Hypericaceae (1)	*** Hypericum perforatum*** L. agg. (3)																
	Flos (2)	2					1		1				1	1			2
	Herba (1)		1					1					1				1
Linaceae (1)	*** Linum usitatissimum*** L. agg. (3)																
	Semen (3)			3		1		2					3				3
Berberidaceae (1)	*** Berberis vulgaris*** L. (3)																
	Herba (3)				3		3						3				3
others (20)	other plant species ^5^ (29)	10	9	18	1	9	8	2	1	5	6	6	28	6		4	38
Total (43)		33	53	111	12	75	63	11	16	13	12	19	165	13	16	15	209

**I** – intact skin; **A** – altered or sore skin; **O** – oral; **QA** – gastrointestinal disorders and metabolic dysfunctions; **QD** – skin afflictions and sores; **QG** – genitourinary system and sex hormones (including peri-partum preparation); **Mast** – mastitis; **QM** – musculoskeletal system (including hematomas and edema in the connective tissue); **GS** – general strengthening; **C** – cattle; **G** – goat; **P** – pigs

^1^Asteraceae (1): *Artemisia absinthium* L. (1); ^2^Lamiaceae (1): *Origanum vulgare* L. agg. (1); ^3^ Rosaceae (3): *Alchemilla mollis* (Buser) Rothm. (2), *Malus domestica* Borkh. (1), *Potentilla anserina* L. (1); ^4^Brassicaceae (1): *Capsella bursa‐pastoris* (L.) Medikus agg. (1); ^5^ Apiaceae (2): *Foeniculum vulgare* Mill. (2), *Carum carvi* L. (1); Araliaceae (1): *Panax ginseng* C.A. Meyer (2); Aspidiaceae (1): *Dryopteris filix‐mas* L. (2); Cannabaceae (1): *Cannabis sativa* L. (2); Caprifoliaceae (1): *Sambucus nigra* L. (1); Ericaceae (1): *Arctostaphylos uva‐ursi* (L.) Sprengel (1); Juglandaceae (1): *Juglans regia* L. (1); Lauraceae (2): *Laurus nobilis* (1), *Cinnamomum camphora* L. (1); Liliaceae (1): *Allium cepa* L. (1); Malvaceae (2): *Althaea officinalis* L. (2), *Malva sylvestris* L. (1); Papaveraceae (1): *Chelidonium majus* L. (1); Pinaceae (2): *Abies alba* Mill. (2), *Picea abies* (L.) H. Karst. (2); Poaceae (1): *Avena sativa* L. (2); Rutaceae (1): *Citrus* x *limon* (L.) Burm.f. (1); Scrophulariaceae (1): *Euphrasia officinalis* L. (2); Xanthorrhoeaceae (1): *Aloe vera* (L.) Burm.f. (1)

^6^ QP (antiparasitic products, insecticides and repellents) agents, QR (respiratory tract) agents, varia

^7^ bees, hens, horses, sheep.

### Further information regarding ItR-UR and GeC-UR

For each UR further information was collected, such as date of last use, frequency of use within the last five years, need for additional therapies, and source of knowledge (Table 5). While in both regions about half of all UR have been used during the last year, 34% of the ItR-UR and 16% of the GeC-UR were used the last time more than 10 years ago.

**Table 5:** Further information on use reports from Italian speaking regions (ItR-UR) and German speaking cantons (GeC-UR) of the study

Information	ItR-UR (total of 205 ItR-UR, percentages in brackets)	GeC-UR (total of 209 GeC-UR, percentages in brackets)
Data of last use		
During last year	102 (50%)	119 (57%)
During last ten years	29 (14%)	49 (23%)
More than 10 years ago	69 (34%)	33 (16%)
Never used, only heard about	3 (1%)	8 (4%)
na	2 (1%)	
Frequency of use within the last five years		
More than 10 times	99 (48%)	78 (37%)
Between 2 and 9 times	11 (5%)	55 (26%)
Once	18 (9%)	17 (8%)
Never	75 (37%)	59 (29%)
Additional therapies in combination		
None	152 (74%)	86 (41%)
Other farmer-administered treatment	47 (23%)	98 (47%)
Treatments by veterinarians	-	17 (8%)
na	6 (3%)	8 (4%)
Source of knowledge		
Ancestors and relatives	131 (64%)	67 (32%)
Friends	39 (19%)	38 (18%)
Books and Journals	15 (7%)	19 (10%)
Courses and Education	6 (3%)	49 (22%)
Personal experience	4 (2%)	34 (16%)
Veterinaries	9 (4%)	-
na	1 (<1%)	2 (1%)
Degree of satisfaction		
n° UR for which it was possible to determinate it on a VAS of 100 mm (average in brackets)	183 (87 mm)	196 (78 mm)

na: information not available

The degree of satisfaction with UR outcomes was measured for 183 ItR-UR and 196 GeC-UR, and average VAS of 87.2 mm and 77.7 mm respectively, were reported (Table 5 and Additional file 1).

## Discussion

Detailed information regarding knowledge and use of homemade herbal remedies from two maximally differing regions of Switzerland (Fig. 1) are reported in our study. ItR is represented by valleys enclosed between the Alps (north) and the Italian border (south). This particular situation leads to cultural differences and similarities both to Switzerland and to Italy. In contrast, GeC may be considered as “in the heart” of the Swiss Confederation. The number of reported plant species named in HSHR is approximately the same for both regions (ItR: 44; GeC: 43), as well as the total number of UR (ItR: 205; GsC: 209).

The aims of our study were (a) to compare the ethnoveterinary practices of two culturally and sociodemographically different regions of Switzerland, ItR and GeC, (b) to analyse to what extent the ethnoveterinary practices in both regions were in accord with earlier studies conducted in neighbouring areas of Switzerland [22, 23] and Italy [24–28] and, (c) to estimate if and how far political borders, language and other reasons could be responsible for regional differences in ethnoveterinary medicine.

### Dialogue partners

Sex and age distribution of dialogue partners in the two study regions were comparable between the research regions and with former Swiss studies (median age 53 [23] and 55 [22] years respectively) but in contrast to dialogue partners in Italian studies (74 [25], 67 [26], and 72 [27] years in average). This may be due to differing methods for recruitment of dialogue partners. However, if this trend will be confirmed in future ethnoveterinary studies, it may be indicative of a higher interest for traditional ethnoveterinary knowledge in the younger generation of Swiss farmers.

Effectiveness of the recruitment methods significantly differed in the two regions, as in ItR 15 out of 25, but only 2 out of 31 interview partners in GeC were recruited via snow-ball sampling [29]. This may be due to cultural differences. In GeC persons apparently felt comfortable in proposing themselves for the project, whereas dialogue partners from ItR seemed more reticent in the beginning. However, after meeting the interviewer in person they were willing to participate, and to help in finding more dialogue partners via their personal contacts. This leads in the end to an overrepresentation of ItR considering the total number of farms of both regions (about 10’000 in GeC and about 1,100 in ItR).

Interviewed farmers in GeC remain after retirement on the farm, while in ItR farms were so small that they often closed with the retirement of the farmer. These small farms have been operated mainly as a second income and were economically unattractive for the following generation. This difference of the two regions might explain also the higher percentage in ItR of remedies used for the last time more than 10 years ago. In ItR the retired farmers have no possibilities to use their knowledge while retired farmers of GeC can assist the following farming generation and transmit their knowledge. One interview in ItR took place just the day after one of Ticino organic farming pioneers sold off his last 4 dairy cows, given that neither relatives nor friends were willing to continue his activity and to pass his knowledge on to a next generation. This example underlines the necessity of ethnoveterinary research for documenting ethnoveterinary practices, in particular, in regions of smallholding farms.

### Origin of used plants for the homemade single-species herbal remedy reports (HSHR)

While in both research regions plant material was purchased in almost 40% of the cases, more cultivated plants were used in GeC (21%; ItR-HSHR, 14%), and plants were slightly more often collected in the wild in ItR (45%; GeC-HSHR, 39%). Commercial products were less used in ItR than in GeC. This may be, in part, explained by the geographical and topographical differences between the two regions, given that ItR is considerably less urbanized and populated. Earlier studies from Switzerland [22] and, even more, from Italy [24–28], reported collection in the wild as the most frequent way for obtaining plants. Regarding the Italian studies methodological differences may, in part, explain the outcomes, given that these studies focused more on botanical aspects (explicitly looking for wild plants), while the focus of Swiss studies were more on veterinary outcomes. However, in Switzerland medicinal plants are more widely available in local pharmacies or druggist stores, while in Italy they are typically only available in the few herbalist shops.

### Extraction procedures for the HSHR

Regarding extraction procedures, in ItR and GeC one third of HSHR were without any extraction. Water extractions were reported frequently in both regions. Infusions appear to be more common in GeC, and decoctions seem to be slightly more common in ItR. Some differences might be directly connected with the commonness of some plant species: e.g. chamomile is typically prepared by infusion, and more common in GeC than in ItR. Other differences might be connected to local cultural aspects: e.g. Coffea spp is prepared as infusion in the northern territories, while in ItR is still reported the old custom of boiling Coffea spp. Former Swiss studies reported similar outcomes regarding extraction procedures, with infusion being the most common mode of extraction, while approximately one third of HSHR were used without any extraction [22, 23]. The same is true for the Italian studies which report a high number of plant species used directly as feed or feed additive, albeit often without obvious medicinal purpose. In contrast, infusions or decoctions appear to be linked to treatment of specific diseases [24–28]. In the perception of the Italian dialogue partners the oral administration of unprocessed plant material is more likely to be a feed additive than a medicine, while some kind of processing or extraction is required to create a “real drug” for the treatment of a disease.

As in former Swiss [22, 23] or Italian studies [24–28] alcohol was less used as extracting agent (“*Schnaps”* (GeC) or “*grappa”* (ItR) with 45 up to 60% vol), normally at room temperature. Exclusively in ItR the use of “*flemma*”, a sub-product of grappa distillation with 16% vol, and decoctions based on red wine and *Syzygium aromaticum* (L.) Merr. & L.M. Perryor *Cinnamonum verum* J. Presl. respectively (“*vin brulè*”) were reported. The latter extraction process was also reported from northern Italy [27].

### Livestock species reported in the use reports (UR) to be treated

Due to the high importance of sheep and goat rearing in ItR, small ruminants were more frequently mentioned in ItR-UR than in GeC-UR (Tables 3 and 4). However, despite the high economic importance of pig and poultry farming and the specific search for dialogue partners involved in this sector in GeC [17], only 4% and 8% of GeC-UR were reported for hens and pigs respectively. In contrast, 8% of ItR-UR were directed towards treatment or prevention of diseases in chicken. This may be related to the low number of chicken per farm kept in ItR compared to GeC, as farmers may be more likely to treat “single” animals than an indistinct large group. Ethnoveterinary remedies seem to be uncommon for the treatment in intensive commercial chicken and pig farms where no individual treatment is possible. These animals were normally treated as a whole herd or flock, but an ethnoveterinary tradition for the treatment of larger herds or flocks (in intensive livestock production) does not exist. This hypothesis is supported by the fact that in European ethnoveterinary studies pigs and poultry are generally less frequently mentioned compared to other animal species such as cattle [2]. This is also true for data from former Swiss [22] and Italian studies [24–28].

### Indications, administration and further information about UR

Disorders against which UR were directed to, were categorized according to the anatomical therapeutic chemical classification system for veterinary medicinal products ATCvet [33] (Fig. 2). In both ItR and GeC, treatments were mainly directed to dermatological (QD) and alimentary tract disorders (QA). These data are consistent with overall data from Europe [2] as well as from Swiss studies [22, 23].

Although differences are reported regarding specific QA pathologies addressed: ItR counts a considerably lower number of UR for treatment of diarrhoea (5%), which is in contrast to GeC-UR (23%), to former Swiss studies [22, 23], and the fact that calf diarrhoea is reported as one of the major health problems in young cattle [36]. In ItR musculoskeletal (QM) and genito-urinary (QG) treatments seem to be more common than in GeC (Fig. 2). In contrast, ItR counts only one UR to treat mastitis, while 13 GeC-UR were mentioned by dialogue partners. However mastitis is considered to be one of major problem in dairy herds [37] and various plant species were reported in previous Swiss [22, 23] and European [2] studies to treat mastitis.

One reason for the differences between both regions might be the high importance of small ruminants in ItR, with a different kind of problems. Antiparasitic treatments, both for endo- and ecto-parasites, are a major concern for ItR farms. These data seem consistent with previous finding which highlighted parasitosis as one of the major issues in sheep and goats [37, 38].

Most of the further information (Table 5) was comparable between both research regions as well as with former Swiss studies [22, 23] but compared to ItR-UR GeC-UR were more frequently reported to be added by another complementary therapy (e.g. homeopathy).

The source of knowledge “Courses and Education” was much more frequently reported for GeC than for ItR (22% versus 3%) and former Swiss studies [22, 23]. This might be due to the higher offer of such courses in GeC through the farmer’s advisory service. The source of knowledge “Ancestors, relatives and friends” was highly overrepresented in ItR-UR (85% versus 50% GeC-UR) and might be a sign for the regional seclusion of ItR. The satisfaction with the outcome of the UR of our both study regions (ItR 87 mm, GeC 78 mm) is comparable to previous Swiss studies which showed values from 80–85 mm in average [22, 23]. None of this further information is available from Italian studies [24–28].

### Drug dosages

Dosages reported by farmers represent their individual empirical knowledge but might be the base for further clinical research, as well as a starting point even for veterinarians to gain own experience [6]. Most of the 27 determined drug dosages of our study (Tables 6 and 7) are well comparable between the both research regions, with previous Swiss studies [22, 23] and human and veterinary literature [39–42] with three exceptions: (1) Coffea spp. is in both research regions applicate in a considerable higher oral dose (ItR: 1.7 g/kg^0.75^; GeC: 1.2 g/kg^0.75^), as in previous Swiss studies (0.4 g/kg^0.75^), (2) *Thymus vulgaris* L. is considerably lower dosed (only determined for GeC: 1.2 g/kg^0.75^) than in one previous Swiss study (2.2 g/kg^0.75^) and (3) for *Urtica dioica* L. was a considerable different dosage determinable in both research region (ItR: 13.8 g/kg^0.75^; GeC: 0.1 g/kg^0.75^) but even between previous Swiss studies some differences and a high diversity was detectable (2.4 and 0.5 g/kg^0.75^). In eight cases we could determine Swiss ethnoveterinary based dosages for the first time (Table 6). Italian data regarding dosages were not available.

**Table 6:** Daily dosage in dry plant equivalent per kg metabolic body weight (g/kg^0.75^) for homemade single species herbal remedy reports (HSHR) with orally administered preparations. Italian speaking regions (ItR), German speaking cantons (GeC), and data from previous Swiss studies [22, 23] are compared, with converted animal dose and converted human dose

Plant species with ≥ 3 reported HSHR and documented dosage	Daily dose [g/kg^0.75^]				
	Arithmetic mean (median; minimum value- maximum value; number of UR for which daily dosage has been estimated)		Determined daily dose [g/kg^0.75^] in previous Swiss studies [22, 23]	Converted animal dose [g/kg^0.75^] [39]	Converted human daily dose [g/kg^0.75^] [40]
	ItR	GeC			
*Achillea millefolium L.* *Flos et herba* cum *flos*	0.25 (0.13; 0.08–0.53; 3)	-	−; −		0.31
*Allium sativum* L. Bulbus	1.09 (0.61; 0.28–3.14; 9)	-	−; 1.12	0.16–0.23	0.09–0.17
*Armoracia rusticana* P. Gaertn. & al. *Radix*	-	2.54 (1.18; 0.26–3.98; 3)	−; −	-	-
*Arnica montana L.* *Herba* cum *radice*	0.012 (0.004; 0.004–0.029; 3)	-	−; −	-	-
*Camellia sinensis* (L.) O. Kuntze. Folium	-	0.38 (0.39; 0.27–0.47; 5)	−; 0.64	1.67–6.67	0.22–0.33
Coffea spp. Semen	1.67 (1.46; 0.31–5.31; 9)	1.19 (0.16; 0.04–9.8; 7)	0.37; 0.35	-	-
*Foeniculum vulgare Mill.* *Semen*	0.07 (0.07; 0.03–0.16; 4)	-	0.24; −	0.2–0.4	0.2–0.3
*Linum usitatissimum* L. Semen	5.79 (3.55; 0.8–15.6; 12)	-	2.92; 5.16	0.39–1.55^ct^ 0.98–1.96^cv^	0.66
*Malva neglecta* Wallr. Folium et herba	0.59 (0.59; 0.29–0.89; 5)	-	−; −	-	-
*Matricaria recutita* L. Flos	0.63 (0.90; 0.18–1.2; 7)	0.27 (0.16; 0.03–0.60; 15)	0.22; 1.12	0.04–0.09 ^p^ 0.19–0.39 ^r^	0.39–0.52
*Matricaria recutita* L. Herba	-	1.32 (1.33; 0.13–2.47; 4)	−; −	-	-
*Oryza sativa* L. Semen	19.79 (17.54; 15.70–26.14; 3)	-	−; −	-	-
*Quercus robur* L. Cortex	-	1.17 (0.94; 0.008–2.82; 5)	−; −	0.19–0.39 ^r^, 0.09–0.19 ^p^	0.13
*Rumex obtusifolius* L. Radix	-	0.84 (0.8; 0.16–1.57; 3)	−; 1.72	-	-
*Thymus vulgaris* L. Herba	-	0.35 (0.25; 0.07–0.98; 9)	2.47; −	0.19–0.39 ^r^	0.04–0.26
*Urtica dioica L.* Herba	13.84 (5.79; 1–41.67; 8)	0.09 (0.08; 0.05–0.16; 4)	2.39; 0.49	0.19–0.39 ^r^	0.35–0.52
*Urtica urens* L. Herba	4.54 (1.00; 0.04–26.6; 11)	-	−; −	-	-

Corresponding average weight in kg/kg metabolic weight per animal species (kg ^0.75^) used in this and previous Swiss studies to calculate daily dosage per kg metabolic weight: Adult cattle 650 kg/128.7 kg ^0.75^; Calf 75 kg/25.5 kg ^0.75^; Goat or Sheep 50 kg 18.8 kg ^0.75^; Young goat or sheep 20 kg 9.5 kg ^0.75^; Pig 200 kg/53.2 kg ^0.75^; Young pig 15 kg/7.6 kg ^0.75^; Horses: 650 kg/128.7 kg ^0.75^; Donkey 200 kg/53.2 kg ^0.75^; Hen 1 kg/1.0 kg ^0.75^; Young chicken: 0.5 kg/0.6 kg ^0.75^; Turkey: 12 kg/6.4 kg ^0.75^; Young turkey:5/3.3 kg ^0.75^; Rabbit 3 kg/2.3 kg ^0.75^; Medium-sized dog 25 kg/11.2 kg ^0.75^; Cat 4 kg/2.8 kg ^0.75^; Rat 0.175 kg/0.3 kg ^0.75^; Human 65 kg/22.9 kg ^0.75^

*ct* cattle, *cv* calves, *r* ruminants, *p* pigs

**Table 7:** Concentration of medicinal plants in homemade single species herbal remedy reports (HSHR) for topical use. Data from Italian speaking regions (ItR), German speaking cantons (GeC), and from earlier Swiss studies [22] are compared with recommended concentrations from literature

Plant species with ≥ 3 reported HSHR and documented dosage	g of dry plant equivalent in 100 g finished product			Recommended concentration g dry plant equivalent in 100 g finished product [39–42]
	Arithmetic mean (median; minimum value- maximum value; number of UR for which concentration has been estimated)		Determined concentration in previous Swiss study [22]	
	ItR	GeC		
*Arnica montana* L. *herba and flos*	1.37 (1.14; 1.00–2.50; 7)	-	2.87	2.0 – 10−33
*Calendula officinalis* L. *herba, flos, and flos sine calice*	2.68 (2.00; 0.48–5.56; 9)	1.10 (0.83; 0.22–3.33; 6)	1.10	0.67– 1.0 – 1.3 – 5.0 – 20 – 50
*Hypericum perforatum* L. herba and flos	2.40 (2.50; 1.85–2.86; 3)	-	1.58	5.0 – 10 – 11
*Malva neglecta* L. *herba, herba* cum *radix, and flos*	0.31 (0.40; 0.13–0.43; 5)	-	0.79	-
*Matricaria recutita* L. *flos*	0.58 (0.50; 0.50–0.75; 3)	1.00 (0.50; 0.15–3.52; 6)	1.52	0.50

### Plant species

**Fig. 3:**
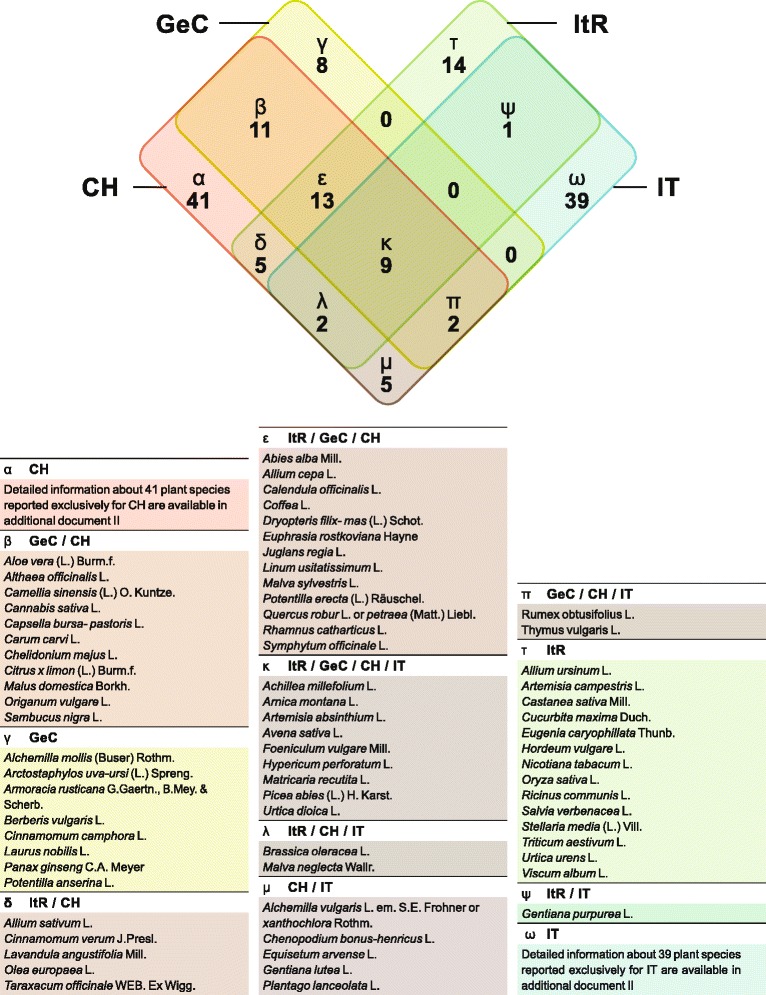
Plant species reported for ethnoveterinary use in both study regions (Italian speaking Region (ItR) and German speaking Cantons (GeC)) compared to earlier ethnoveterinary studies conducted in northern Italy (IT) [24–28] and Switzerland (CH) [22, 23]

Since ethnoveterinary data from Italy [24–28] were collected with different methodologies than in Switzerland we decided to focus on the reported plant species to highlight similarities and differences of the different regions (Fig. 3 and Additional file 2). GeC as well as ItR shared about one quarter of the reported plant species with the Italian studies while four fifth (GeC) and two thirds (ItR) are in accordance with former Swiss studies [22, 23]. A comparison with German ethnoveterinary data would have been of interest but was not possible because such data are not available.

However, the comparison of Swiss and Italian ethnoveterinary data shows a higher similarity than the comparison of Romanian with Spanish, Italian and Albanian data [13], but it is comparable with the overlapping of southern Italian with Tunisian data [15]. This suggests that ethnoveterinary tradition may change with growing geographic distance.

Interestingly, no plant species of GeC corresponds exclusively to a plant species of ItR or Italy but about one quarter of the GeC plant species corresponds exclusively with former Swiss studies. In contrast only one ItR plant species overlaps exclusively with Italian (*Gentiana purpurea* L., which also shares indication [26]) but 5 exclusively with former Swiss studies. To our surprise ItR data are closer to former Swiss [22, 23] than to Italian studies, even though we included explicitly only studies of mountain regions of northern Italy [24–28]. However, with one third of the plant species being exclusively reported from ItR compared to only one fifth from GeC, ItR shows a slightly higher independence in this aspect than GeC. Most of the exclusively reported plant species are cited only with one UR. Three or more UR are reported only for *Oryza sativa* L. and *Urtica urens* L. (ItR-UR) and *Armoracia rusticana* P. Gaertn. & al. and *Berberis vulgaris* L. (GeC-UR). *U. urens* L. is one of the most often reported species in ItR, while only a few European ethnobotanic surveys report its use to treat animals [43–45]. Four plant species each of ItR (*Allium ursinum* L., *Artemisia campestris* L., *Cucurbita maxima* Duch. and *Eugenia caryophillata* Thunb.) and GeC (*Alchemilla mollis* (Buser) Rothm., *Armoracia rusticana* P. Gaertn. & al., *Cinnamomum camphora* (L.) J.Presl. and *Panax ginseng* C.A. Mayer) have never been cited before in European ethnoveterinary literature to treat animal diseases [2].

About half of the plant species (22 species, Fig. 3) were named in both research regions. Some of them in the context of the same indications: e.g. *Matricaria recutita* L. is used in both regions to treat gastrointestinal as well as dermatological disorders. However, ItR and GeC share less than half of the plant species representing their usualness with more than 4 UR (Table 8): e.g. Malvaceae family, well known from previous Swiss [22, 23] and European studies [2] as a dermatological agent, is apparently well known only in ItR. Information regarding the frequency of use is not available from Italian studies [24–28]. However, other studies show bigger differences in ethnoveterinary tradition even from neighbouring regions of the same linguistic area in the same country [14]. In that respect Switzerland shows a relative high consensus in ethnoveterinary data.

**Table 8:**
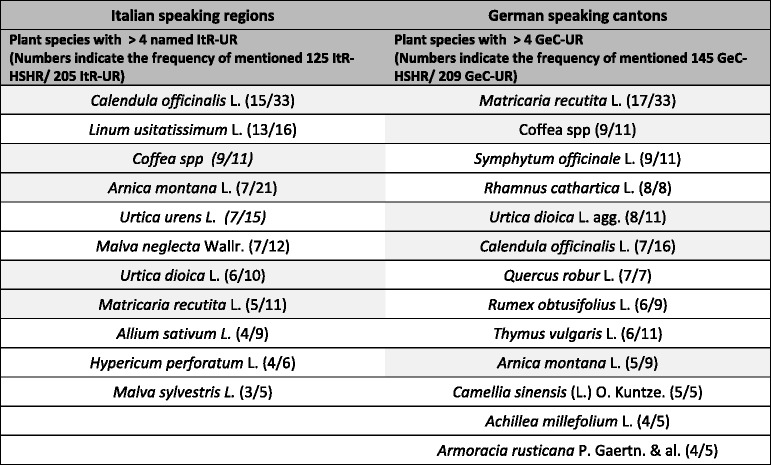
Plant species most frequently reported in the two regions of the study

Plant species used in both regions are highlighted in grey

*HSHR* Homemade single species remedy report, *UR* use reports

Natural conformation of the territories may explain at least some of the major differences between the two areas of the same country: for example *Arnica montana* L. naturally grows in the alpine regions and thus probably is associated with a higher number of ItR-UR than GeC-UR. Another example may be *Urtica urens* L. use in ItR, where is preferred to *Urtica dioica* L., probably because of its local prevalence on country side.

The attitude towards phytotherapy (for humans) is generally positive in Switzerland [46], and research in the field has been actively pursued over the last decades [47]. Availability of herbal drugs and finished products is therefore rather good. In contrast, phytotherapy is much less popular in Italy, and most of the plants used for veterinary purposes were harvested from the wild. This may, at least in part, explain the differences in the spectrum of plant species reported in the ethnoveterinary surveys. Nevertheless, how much differing policies and attitudes regarding medicinal plants are responsible for these national differences remains an open question. Different farm structure and main livestock species and, hence, different major pathologies likely also contributed to these differences. However, geographical and linguistic barriers on the one side, and political borders on the other side seem to contribute to the relative uniqueness of ItR ethnoveterinary knowledge, whereby political borders seem to be a higher barrier than mountain ranges.

## Conclusion

We conducted the first ethnoveterinary study comparing two culturally and socio-demographically differing regions of Switzerland. They differ in language, as well as in history, culture, agricultural structure, and topography. Dialogue partners of ItR were Italian speaking smallholder farmers in a mountainous region. Most of them kept small ruminants and obtained their knowledge mainly from ancestors and friends. Dialogue partners of GeC were Swiss-German speaking farmers of lowland regions. They possessed larger farms, and many of them kept pigs and poultry. These farmers obtained their knowledge more often from courses and education. However, most of the farmers of ItR and GeC kept cattle and were comparable with respect to age and gender distribution.

Farmers from both ItR and GeC of Switzerland know and currently use homemade herbal remedies to treat different livestock species. We documented in our survey a wide spectrum of plant species (44 in ItR, 43 in GeC; 65 in total), preparations (125 ItR-HSHR and 145 GeC-HSHR), and use reports (205 ItR-UR, 209 GeC-UR). About half of the cited plant species of GeC and ItR were the same. Compared to GeC and to earlier studies from Switzerland and adjacent Italian regions, ItR showed some uniqueness regarding the reported plant species but, surprisingly, nearly no exclusive similarities with Italian data. Therefore, ItR patterns of ethnoveterinary use of plant species seem closer to the rest of Switzerland than to northern Italy. Based on our findings we conclude that linguistic, geographical, environmental as well as social and farm-structural conditions influence the regional ethnoveterinary knowledge, but that political borders are more important than language or geographical barriers. For this reason the documentation of ethnoveterinary knowledge from other European countries is urgently needed, in particular from central, northern and eastern Europe where no data are currently available.

## Additional files

Additional file 1: Use reports of Swiss farmers in a) Italian speaking regions and b) north western German speaking Cantons. (PDF 185 kb)
Additional file 2: List of all reported plant species used to treat animals in West Alps regions. (PDF 139 kb)

## Abbreviations

GeC: German speaking canton
HRWP: Homemade remedy reports without plant ingredients
HSHR: Homemade single-species herbal remedy report
ItR: Italian speaking region
UR: Use report
VAS: Visual analogue scale
