# Supplementary material for: Swiss ethnoveterinary knowledge on medicinal plants – a within-country comparison of Italian speaking regions with north-western German speaking regions
Source: J Ethnobiol Ethnomed. 2017 Jan 3;13:1. doi: 10.1186/s13002-016-0106-y (PMC5209851; doi:10.1186/s13002-016-0106-y)
Supplement: Additional file 1: — Use reports of Swiss farmers in a) Italian speaking regions and b) north western German speaking Cantons. (PDF 185 kb) [file 13002_2016_106_MOESM1_ESM.pdf]

Additional Document I : Ethnoveterinary herbal remedies used by farmers in a) Italian speaking Regions (Recepy number starts with 1717; Dialogue partner number starts with 203)

One line in the following table represents one use report: [dialogue partner] x [plant species] x [plant part] x [manufacturing process to the finished product] x [category of use] x [specification of use] x[animal species] x [animal age classification] x [administration procedure]. The [specification of use] is not included in the additional file 1 for space reasons. This explains identical lines.

List of abbreviations

General:
na= information not available

Plant part:
bar= bark
exc= excretions
flo= flowers and inflorescenses
fsb= fruits, seeds, berries
her= whole plants without roots (herb)
lea= leaves
pet= petals
rob= root/ bulb
twb= twigs, branches
wpr= whole plants with roots

Conc. [g/100g]
Concentration [g drug in 100g finished product]; used in formulations for topical treatment and intravaginal/ intrauterine administration;
da= directly administered without extraction, external administration
tohe= treatment of housing environment

Origin:
bo= bought/ crude drug
bo*= bought/ commercial extracts and finished products
cu= cultivated
re= received by another local farmer
wh= wild harvesting

(vs)= voucher specimen available
(vs*)= voucher specimen available from former Swiss ethnoveterinary research [22]

VAS: visual analogue scale in mm (0 mm corresponding with “no effect”, 100 to “very good effect”)

Animal treated:
nsas= no specification of the animal species (external administration)

Extraction procedure on farm:
none
alcohol: (rt)= room temperature
(et)= elevated temperature
milk: (rt)= room temperature
(et)= elevated temperature
oil/fat: (rt)= room temperature
(et)= elevated temperature
water : (rt)=room temperature
(dec)=decoction
(inf)= infusion

Daily dosage [$g/kg^{0.75}$]
in plant equivalent per kg metabolic body weight [$g/kg^{0.75}$]; used in formulations for oral administration only;

Categories of use:
QA= gastrointestinal disorders and metabolic dysfunctions;
QD = skin afflictions and sores;
QG = genito-urinary system and sex hormones (including peri-partum preparation);
Mast = mastitis;
QM = musculoskeletal system (including hematomas and oedema in the connective tissue);
QP = antiparasitics products, insecticides and repellents;
QR = respiratory tract agents;
QS = sensory organs agents;
QN/behav = nervous system agent and behaviour;
Varia/GS = various conditions/general strengthen

Ver= Verification of dosage
ew= estimated weight by assessment of the volume and subsequent weighing
od= original drug weighted on- site
rd= reference drug weighted on- site

Administration
exal= external administration, altered or sore skin
(epicutan)= epicutaneous
(conj)= conjunctival
(hoof)= skin appendages - hoofs
(teud)= skin on teats and udder
(navel)
exin= external administration, intact skin
(epicutan)= epicutaneous
(conj)= conjunctival
(teud)= skin on teats and udder
(hoof)= skin appendages – hoofs
int= internal administration
(nasal)= nasal
(oral)= oral
(vaut)= intravaginal/ intrauterine
tohe= treatment of housing environment

RN= Recipe number
DP= Dialog partner

Botanical family	Plant species	Recepy name designated by DP	Plant part	Origin	Extraction procedure on farm	ATCvet code	Animal species treated	Administration	Daily dosage (g/kg$^{0.75}$)	Conc. (g/100g)	Ver	VAS	RN	DP
**Amaryllidaceae**	*Allium ursinum* L.	aglio orsino	lea	wh	none	QA	hen	Int (oral)	2,1		ew	93	1722	203
**Apiaceae**	*Foeniculum vulgare* Mill.	finocchio	fsb	bo	water (inf)	QA	young sheep	Int (oral)	0,17		ew	3	1785	213
		finocchio	fsb	bo	water (inf)	QA	dog	Int (oral)	0,03		ew	97	1804	214
		finocchio	fsb	bo	water (inf)	QA	young goat	Int (oral)	0,04		ew	21	1804	214
		finocchio	fsb	bo	water (inf)	QA	cat	Int (oral)	0,06		ew	98	1804	214
**Aspidiaceae**	*Dryopteris filix-mas* L. (Schott) (vs)	felce	her	wh	none	QP	sheep	Int (oral)	na		na	97	1833	219
**Asteraceae**	*Achillea millefolium* L. (vs)	millefoglie	her	wh	water (inf)	QG peri-partum	cattle	Int (oral)	0,08		rd	81	1814	217
		millefoglie	her	wh	water (inf)	QG peri-partum	goat	Int (oral)	0,21		rd	51	1814	217
		achillea	flo	wh	water (inf)	QA	sheep	Int (oral)	0,13		ew	100	1831	219
	Arnica montana L.	arnica	wpr	wh	alcohol (rt)	QM	goat	exin (epicutan)		1	od	100	1736	204
		arnica	wpr	wh	alcohol (rt)	QM	cattle	exin (epicutan)		1	od	100	1736	204
		arnica	wpr	wh	alcohol (rt)	QM	cattle	Int (oral)		1	od	100	1736	204
		arnica	wpr	wh	alcohol (rt)	QG peri-partum	horse	exin (teud)		5,56	od	100	1736	204
		arnica	wpr	wh	alcohol (rt)	QM	horse	exin (epicutan)		na	od	100	1736	204
		arnica	wpr	wh	alcohol (rt)	QM	horse	Int (oral)	na		od	100	1736	204
		arnica	wpr	wh	alcohol (rt)	QM	goat	Int (oral)	na		od	100	1736	204
		tintura di arnica	flo	wh	alcohol (rt)	QD	sheep, young sheep	exal (epicutan)		1,36	ew	96	1812	217
		tintura di arnica	flo	wh	alcohol (rt)	QD	goat, young goat	exal (epicutan)		1,36	ew	95	1812	217
		tintura di arnica	flo	wh	alcohol (rt)	QD	young goat	exal (navel)		1,36	ew	96	1812	217
		tintura di arnica	flo	wh	alcohol (rt)	QD	young sheep	exal (navel)		1,36	ew	97	1812	217
		tintura di arnica	flo	wh	alcohol (rt)	QD	calf	exal (navel)		na	ew	96	1812	217
		arnica	flo	wh	alcohol (rt)	QM	cattle, calf	exin (epicutan)		1,36	ew	97	1823	218
		arnica tintura	flo	wh	alcohol (rt)	QD	sheep, young sheep	exal (teud)		1,11	ew	96	1834	219
		arnica in acqua	flo	wh	water (rt)	QD	sheep, young sheep	exal (teud)		1,14	ew	95	1835	219
		arnica	flo	wh	alcohol (rt)	QM	cattle	exin (epicutan)		1,11	ew	93	1839	220
		arnica	flo	wh	alcohol (rt)	QM	cattle, calf	exin (teud)		1,11	ew	95	1839	220
		arnica	flo	wh	alcohol (rt)	QM	goat, young goat	exin (epicutan)		1,11	ew	95	1839	220
		arnica	flo	wh	alcohol (rt)	QM	goat, young goat	exin (teud)		1,11	ew	94	1839	220
		arnica	flo	wh	alcohol (rt)	QM	horse	exin (epicutan)		2,5	ew	88	1872	225
		arnica	flo	wh	alcohol (rt)	QM	sheep, young sheep	exin (epicutan)		2,5	ew	89	1872	225

Botanical family	Plant species	Recepy name designated by DP	Plant part	Origin	Extraction procedure on farm	ATCvet code	Animal species treated	Administration	Daily dosage (g/kg$^{0.75}$)	Conc. (g/100g)	Ver	VAS	RN	DP
**Asteraceae**	*Artemisia absinthium* L. (vs)	erba bianca	her	wh	none	QP	pig, piglets	tohe		tohe	na	98	1842	222
	Artemisia campestris L.	artemisia beifus	rob	wh	water (dec)	QG peri-partum	goat	Int (oral)	0,53		rd	na	1782	212
	Calendula officinalis L.	flemma e calendula	flo	cu	alcohol (rt)	QM	cattle, calf	exin (epicutan)		2	od	70	1717	203
		flemma e calendula	flo	cu	alcohol (rt)	QD	dog	exal (epicutan)		2	od	92	1717	203
		Calendula olio	flo	cu	oil/fat (rt)	QM	goat, young goat	exin (epicutan)		5,55	ew	99	1734	204
		Calendula olio	flo	cu	oil/fat (rt)	QM	cattle, calf	exin (epicutan)		5,56	ew	99	1734	204
		Calendula olio	flo	cu	oil/fat (rt)	QM	sheep, young sheep	exin (epicutan)		5,56	ew	99	1734	204
		Calendula olio	flo	cu	oil/fat (rt)	QM	horse	exin (epicutan)		5,56	ew	99	1734	204
		Calendula olio	flo	cu	oil/fat (rt)	QD	goat	exal (epicutan)		5,56	ew	99	1734	204
		la calendula	flo	cu	oil/fat (rt)	QD	goat, young goat	exal (teud)		2	ew	88	1777	212
		calendula tintura	na	bo*	none	QD	goat	exal (hoof)		na	na	85	1778	212
		calendula crema	flo	cu	oil/fat (et)	QD	sheep	exal (navel)		na	ew	89	1789	213
		crema di calendula	flo	bo*	none	QD	goat	exal (teud)		na	na	100	1802	214
		calendula tintura	flo	cu	alcohol (rt)	QD	sheep, young sheep	exal (epicutan)		1,43	ew	99	1813	217
		calendula tintura	flo	cu	alcohol (rt)	QD	goat, young goat	exal (epicutan)		1,43	ew	100	1813	217
		calendula tintura	flo	cu	alcohol (rt)	QD	cattle, calf	exal (epicutan)		1,43	ew	na	1813	217
		crema di calendula	flo	cu	oil/fat (et)	QD	goat, young goat	exal (epicutan)		1,2	ew	100	1819	217
		crema di calendula	flo	cu	oil/fat (et)	QD	sheep, young sheep	exal (epicutan)		1,2	ew	100	1819	217
		crema di calendula	flo	cu	oil/fat (et)	QD	cattle, calf	exal (epicutan)		1,2	ew	98	1819	217
		calendula	flo	bo*	none	QD	cattle, calf	exal (epicutan)		da	na	91	1822	218
		calendula pomata	flo	cu	oil/fat (et)	QD	sheep	exal (teud)		na	na	97	1836	219
		calendula pomata	flo	cu	oil/fat (et)	QM	sheep, young sheep	exin (epicutan)		na	na	93	1836	219
		calendula pomata	flo	cu	oil/fat (et)	QD	goat	exal (teud)		0,48	ew	92	1840	220
		calendula pomata	flo	cu	oil/fat (et)	QD	cattle	exal (teud)		0,48	ew	92	1840	220
		calendula pomata	her	cu	oil/fat (et)	QD	young sheep	exal (epicutan)		3,85	ew	87	1844	222
		calendula pomata	her	cu	oil/fat (et)	QD	sheep, young sheep	exal (epicutan)		3,85	ew	77	1844	222
		calendula pomata	her	cu	oil/fat (et)	QD	cattle, calf	exal (epicutan)		3,85	ew	87	1844	222
		calendula tintura	flo	bo*	alcohol (rt)	QD	cattle, calf	exal (epicutan)		na	ew	99	1860	223
		calendula tintura	flo	bo*	alcohol (rt)	QD	sheep, young sheep	exal (epicutan)		na	ew	98	1860	223
		calendula tintura	flo	bo*	alcohol (rt)	QD	cattle	exal (teud)		na	ew	98	1860	223

Botanical family	Plant species	Recepy name designated by DP	Plant part	Origin	Extraction procedure on farm	ATCvet code	Animal species treated	Administration	Daily dosage (g/kg$^{0.75}$)	Conc. (g/100g)	Ver	VAS	RN	DP
**Asteraceae**	*Calendula officinalis* L.	calendula tintura	flo	bo*	alcohol (rt)	QD	sheep	exal (teud)		na	ew	98	1860	223
		calendula	flo	bo*	alcohol (rt)	QD	cattle, calf	exal (epicutan)		na	ew	99	1861	224
		crema di calendula weleda	flo	bo*	none	QD	horse	exal (epicutan)		na	na	90	1871	225
		crema di calendula weleda	flo	bo*	none	QD	dog	exal (hoof)		na	na	95	1871	225
		crema di calendula weleda	flo	bo*	none	QD	horse	exal (epicutan)		na	na	95	1871	225
	Matricaria recutita L. (vs*)	la camomela	flo	cu	water (inf)	QD	cattle	Int (oral)	0,18		rd	53	1755	209
		la camomela	flo	cu	water (inf)	QD	calf	Int (oral)	na		rd	53	1755	209
		camomilla	flo	bo	water (inf)	QS	young sheep	exal (conj)		0,75	rd	50	1768	211
		camomilla	flo	cu	water (inf)	QA	cattle	Int (oral)	0,29		rd	80	1816	217
		camomilla	flo	cu	water (inf)	QA	sheep	Int (oral)	1,2		rd	66	1816	217
		camomilla	flo	cu	water (inf)	QA	goat	Int (oral)	1,2		rd	100	1816	217
		camomilla	flo	cu	water (dec)	QD	calf	exal (navel)		0,5	rd	95	1829	218
		camomilla	flo	cu	water (dec)	Varia/GS	horse	exin (epicutan)		0,5	rd	93	1829	218
		camomilla	flo	cu	water (dec)	QD	cattle, calf	exal (epicutan)		0,5	rd	92	1829	218
		camomilla	flo	cu	water (dec)	QA	cattle	Int (oral)	na		rd	90	1829	218
		camomilla	flo	wh	water (inf)	QA	young goat	Int (oral)	0,21		rd	47	1858	223
	Taraxacum officinale Weber Ex F.H. Wigg.	Tarassaco tintura	her	bo*	none	QA	sheep	Int (oral)	na		od	87	1765	211
		Tarassaco tintura	her	bo*	none	QD	horse	Int (oral)	na		od	90	1765	211
		Tarassaco	wpr	wh	alcohol (rt)	QD	horse	Int (oral)	0,04		ew	80	1766	211
		Tarassaco	wpr	wh	alcohol (rt)	QA	sheep	Int (oral)	0,27		ew	88	1766	211
**Boraginaceae**	*Symphytum officinale* L. (vs*)	Consolida	rob	wh	none	QM	goat	exin (epicutan)		da	na	93	1781	212
**Brassicaceae**	*Brassica oleracea L.* (convar. Capitata var. Sabauda L.)	foglie di cavolo verza	lea	cu	none	QD	cattle, calf	exal (epicutan)		da	od	83	1748	209
**Caryophyllaceae**	*Stellaria media* (L.) Vill. (vs)	erba di gallin o peverina	her	cu	none	Varia/GS	hen	Int (oral)	20		od	94	1724	203
		paverina	her	wh	none	Varia/GS	hen	Int (oral)	16		ew	100	1818	217
**Cucurbitaceae**	*Cucurbita maxima* Duch.	semi di zucca	fsb	cu	none	QP	young goat	Int (oral)	na		na	na	1770	211
		semi di zucca	fsb	cu	none	QP	goat	Int (oral)	na		na	na	1770	211
**Euphorbiaceae**	*Ricinus communis* L.	olio di ricino	fsb	bo*	none	QA	young sheep	Int (oral)	na		na	99	1788	213
**Fagaceae**	*Castanea sativa* Mill.	La corteccia del castagno	bar	wh	none	QP	goat	Int (oral)	na		na	na	1769	211
		la corteccia del castagno	bar	wh	none	QP	young goat	Int (oral)	na		na	na	1769	211
	Quercus robur L.	corteccia di quercia	bar	wh	water (dec)	QA	young goat	Int (oral)	3,51		ew	87	1739	205
**Gentianaceae**	*Gentiana purpurea* L.	genziana	rob	wh	none	QA	cattle	Int (oral)	0,05		na	81	1754	209

Botanical family	Plant species	Recepy name designated by DP	Plant part	Origin	Extraction procedure on farm	ATCvet code	Animal species treated	Administration	Daily dosage (g/kg$^{0.75}$)	Conc. (g/100g)	Ver	VAS	RN	DP
**Gentianaceae**	*Gentiana purpurea* L.	genziana	rob	wh	water (dec)	QA	cattle	Int (oral)	0,23		rd	96	1824	218
**Hypericaceae**	*Hypericum perforatum* L. (vs)	Iperico	her	wh	oil/fat (rt)	QD	nsas	exal (epicutan)		1,85	ew	100	1737	204
		Iperico	her	wh	oil/fat (rt)	QN	goat	Int (oral)	na		ew	45	1737	204
		olio di iperico	flo	re	oil/fat (rt)	Mast	sheep	exin (teud)		na	na	97	1832	219
		olio rosso	flo	wh	oil/fat (rt)	QD	young sheep	exal (epicutan)		2,86	ew	99	1841	221
		olio rosso	flo	wh	oil/fat (rt)	QD	sheep	exal (teud)		2,86	ew	82	1841	221
		olio di san giovanni	flo	wh	oil/fat (rt)	QM	horse	exin (epicutan)		2,5	ew	54	1873	225
**Juglandaceae**	*Juglans regia* L.	noce	lea	wh	none	QP	dog	exin (epicutan)		da	na	98	1846	223
**Lamiaceae**	*Lavandula angustifolia* Mill.	la lavanda	wpr	cu	none	QP	nsas	tohe		tohe	od	na	1773	211
	Salvia verbenacea L.	salvia selvatica	lea	wh	none	QD	cattle	exal (epicutan)		da	od	95	1751	209
**Lauraceae**	*Cinnamomum verum* J.PRESL	vin brulè	bar	bo	alcohol (et)	QA	cattle	Int (oral)	na		na	na	1825	218
**Liliaceae**	*Allium cepa* L.	sciroppo per la tosse alla cipolla	rob	bo	honey (rt)	QR	horse	Int (oral)	0,19		ew	89	1868	225
	Allium sativum L.	aglio	rob	bo	none	QP	turkey	Int (oral)	0,27		na	96	1720	203
		aglio	rob	bo	none	QP	turkey	Int (oral)	0,27		na	96	1720	203
		aglio	rob	bo	none	QP	hen	Int (oral)	1,7		na	95	1720	203
		aglio	rob	bo	none	QP	hen	Int (oral)	1,7		na	96	1720	203
		aglio	rob	bo	none	QP	cattle	Int (oral)	1,24		od	99	1760	210
		aglio	rob	bo	none	QP	calf	Int (oral)	3,14		od	99	1760	210
		aglio	rob	bo	none	QP	goat	Int (oral)	0,54		ew	76	1800	214
		aglio	rob	bo	none	QP	dog	Int (oral)	0,3		ew	na	1843	222
		aglio	rob	bo	none	QP	cat	Int (oral)	0,61		ew	75	1843	222
**Linaceae**	*Linum usitatissimum* L.	cura rinfrescante	fsb	bo	none	Varia/GS	cattle	Int (oral)	na		na	98	1726	203
		linosa	fsb	bo	water (rt)	QG peri-partum	cattle	Int (oral)	3,66		rd	80	1727	203
		linosa	fsb	bo	water (rt)	QG peri-partum	pig	Int (oral)	8,85		rd	79	1727	203
		semi di lino	fsb	bo	water (rt)	QA	horse	Int (oral)	3,89		rd	83	1728	204
		shmellin	fsb	bo	water (rt)	QG peri-partum	cattle	Int (oral)	3,16		rd	98	1744	207
		shmellin	fsb	bo	water (rt)	Varia/GS	cattle	Int (oral)	3,16		rd	99	1744	207
		shmellin	fsb	bo	water (rt)	Varia/GS	cattle	Int (oral)	3,15		rd	79	1745	205
		shmellin	fsb	bo	water (rt)	Varia/GS	goat	Int (oral)	9,97		rd	76	1745	205
		linosa	fsb	bo	water (rt)	QG peri-partum	cattle	Int (oral)	3,45		rd	73	1757	209
		semi di lino	fsb	bo	none	QG peri-partum	goat	Int (oral)	na		rd	na	1774	211

Botanical family	Plant species	Recepy name designated by DP	Plant part	Origin	Extraction procedure on farm	ATCvet code	Animal species treated	Administration	Daily dosage (g/kg$^{0.75}$)	Conc. (g/100g)	Ver	VAS	RN	DP
**Linaceae**	*Linum usitatissimum* L.	semi di lino	fsb	bo	none	QG peri-partum	sheep	Int (oral)	na		na	na	1787	213
		linosa	fsb	bo	water (rt)	QG peri-partum	goat	Int (oral)	15,96		rd	100	1797	214
		semi di lino	fsb	bo	water (rt)	QG peri-partum	goat	Int (oral)	1,6		rd	85	1817	217
		semi di lino	fsb	bo	water (dec)	QG peri-partum	cattle	int (vaut)	0,19		rd	96	1827	218
		linosa	fsb	bo	water (rt)	Varia/GS	cattle	Int (oral)	0,8		rd	77	1865	223
		semi di lino	fsb	bo	water (rt)	QA	horse	Int (oral)	0,13		ew	91	1869	225
**Loranthaceae**	*Viscum album* L. S.L.	vischio	her	wh	none	QG	nsas	tohe		tohe	na	na	1772	211
**Malvaceae**	*Malva neglecta* Wallr. (vs*)	la malva selvatica	lea	wh	water (inf)	QA	goat	Int (oral)	1,77		ew	96	1741	205
		la malva selvatica	lea	wh	water (inf)	QA	calf	Int (oral)	4,57		ew	95	1741	205
		malva domestica	her	wh	water (inf)	QD	goat	exal (hoof)	0,05		rd	30	1779	212
		malva domestica	her	wh	water (inf)	QD	goat	Int (oral)	na		rd	na	1779	212
		malva	lea	wh	water (inf)	QD	cattle	exin (hoof)		0,43	rd	86	1806	215
		malva	lea	wh	water (dec)	QG peri-partum	cattle	int (vaut)	0,03		rd	99	1826	218
		malva	her	wh	water (inf)	QD	cattle, calf	exal (hoof)		0,13	rd	72	1838	220
		acqua malva	wpr	wh	water (dec)	QD	calf	exal (navel)		0,40	rd	74	1862	224
		acqua malva	wpr	wh	water (dec)	QD	cattle	exal (teud)		0,4	rd	74	1862	224
		acqua malva	wpr	wh	water (dec)	QD	calf	exal (navel)		0,40	rd	74	1863	223
		acqua malva	wpr	wh	water (dec)	QD	cattle	exal (teud)		0,4	rd	75	1863	223
		acqua malva	wpr	wh	water (dec)	QD	cattle, calf	exal (epicutan)		0,4	rd	75	1863	223
	Malva sylvestris L.	la malva domestica	lea	wh	water (inf)	QA	goat	Int (oral)	1,77		ew	96	1740	205
		la malva domestica	lea	wh	water (inf)	QA	calf	Int (oral)	4,57		ew	95	1740	205
		malva	her	wh	water (dec)	QD	cattle	exal (epicutan)		2,5	rd	63	1750	209
		malva	her	wh	water (dec)	QD	horse	exal (epicutan)		2,5	rd	61	1750	209
		malva selvatica	her	wh	water (inf)	QD	cattle, calf	exal (hoof)		0,13	rd	73	1877	220
**Myrtaceae**	*Eugenia caryophyllata* Thunb.	vin brulè	flo	bo	alcohol (et)	QG peri-partum	cattle	Int (oral)	na		na	na	1759	210
**Oleaceae**	*Olea europaea* L.	olio sale e aceto	fsb	bo	none	QA	sheep	Int (oral)	na		od	100	1786	213
		olio d'oliva	fsb	bo	none	QD	calf	exin (epicutan)		da	ew	89	1864	224
**Pinaceae**	*Abies alba* Mill.	resina	exc	wh	none	QP	goat	Int (oral)	na		na	99	1733	204
		resina	exc	wh	none	QP	young goat	Int (oral)	na		na	99	1733	204
	Picea abies (L.)H. Karst.	labbiot	exc	wh	none	QM	goat, young goat	exin (epicutan)		da	na	100	1821	218
		resina	exc	wh	none	QD	sheep	exal (hoof)		da	na	99	1837	219

Botanical family	Plant species	Recepy name designated by DP	Plant part	Origin	Extraction procedure on farm	ATCvet code	Animal species treated	Administration	Daily dosage (g/kg$^{0.75}$)	Conc. (g/100g)	Ver	VAS	RN	DP
**Pinaceae**	*Picea abies* (L.)H. Karst.	resina	exc	wh	none	QM	sheep, young sheep	exal (epicutan)		da	na	99	1837	219
		resina di abete	exc	wh	none	QM	nsas	exal (epicutan)		da	na	93	1850	223
**Poaceae**	*Avena sativa* L.	avena	fsb	bo	none	QG	goat	Int (oral)	5,32		ew	97	1801	214
	Hordeum vulgare L. S.L.	orzo	fsb	bo	none	QG peri-partum	goat	Int (oral)	5,31		od	100	1798	214
	Oryza sativa L.	riso bollito	fsb	bo	water (dec)	QA	calf	Int (oral)		16,66	od	75	1718	203
		acqua del riso	fsb	bo	water (dec)	QA	calf	Int (oral)	15,7		od	94	1763	210
		acqua di riso	fsb	bo	water (dec)	QA	young goat	Int (oral)	17,54		ew	79	1859	223
		acqua di riso	fsb	bo	water (dec)	QA	calf	Int (oral)	26,14		ew	79	1859	223
	Triticum aestivum L.	laitada	fsb	bo	water (inf)	QG peri-partum	cattle	Int (oral)	15,54		ew	50	1876	227
**Rhamnaceae**	*Rhamnus catharticus* L. (vs)	kreuzdorn	twb	cu	none	QD	calf	tohe		tohe	na	94	1845	222
		kreuzdorn	twb	cu	none	QD	cattle, calf	tohe		tohe	na	na	1874	226
**Rosaceae**	*Potentilla erecta* (L.) Räuschel (vs*)	potentilla o tormentilla	her	bo	none	QA	calf	Int (oral)	na		na	94	1780	212
**Rubiaceae**	Coffea spp.	caffè con vino	fsb	bo	water (dec)	QG peri-partum	cattle	Int (oral)	1,55		rd	91	1742	205
		caffè con vino	fsb	bo	water (dec)	QG peri-partum	goat	Int (oral)	5,32		rd	92	1742	205
		caffè con grappa	fsb	bo	water (dec)	QA	cattle	Int (oral)	0,93		od	na	1743	206
		caffè con grappa	fsb	bo	water (dec)	QA	cattle	Int (oral)	0,31		rd	60	1753	209
		un intruglio di caffè e vino	fsb	bo	water (dec)	QG	cattle	Int (oral)	na		na	na	1771	211
		grappa e caffè	fsb	bo	water (inf)	QG peri-partum	goat	Int (oral)	2,47		od	100	1793	214
		caffè solo o con grappa	fsb	bo	water (inf)	QA	young sheep	Int (oral)	0,79		ew	95	1830	219
		caffè solo o con grappa	fsb	bo	water (inf)	QA	sheep	Int (oral)	0,8		ew	97	1830	219
		caffè e grappa	fsb	bo	water (dec)	QA	goat	Int (oral)	1,46		rd	52	1866	223
		caffè e grappa	fsb	bo	water (dec)	QA	goat	Int (oral)	1,46		rd	na	1867	224
		caffè e grappa	fsb	bo	water (dec)	QA	cattle	Int (oral)	na		na	88	1875	227
**Scrophulariaceae**	*Euphrasia rostkoviana* Hayne	eufrasia Augentrost	her	wh	water (inf)	QS	goat	exal (conj)		0,96	ew	95	1775	212
		eufrasia	her	wh	water (inf)	QS	cat	exal (conj)		1,2	ew	92	1849	224
**Solanaceae**	*Nicotiana tabacum* L.	sigarette	lea	bo	water (rt)	QP	cattle, calf	exin (epicutan)		0,02	ew	91	1795	214
	Nicotiana tabacum L.	sigarette	lea	bo	water (rt)	QP	goat, young goat	exin (epicutan)		0,02	ew	89	1795	214
**Urticaceae**	*Urtica dioica* L.	ortica	her	wh	none	Varia/GS	turkey	Int (oral)	3,9		od	93	1723	203
		ortica	her	wh	none	Varia/GS	young turkey	Int (oral)	7,58		od	93	1723	203
		ortica	her	wh	none	Varia/GS	hen	Int (oral)	25		od	95	1723	203

Botanical family	Plant species	Recepy name designated by DP	Plant part	Origin	Extraction procedure on farm	ATCvet code	Animal species treated	Administration	Daily dosage (g/kg$^{0.75}$)	Conc. (g/100g)	Ver	VAS	RN	DP
**Urticaceae**	*Urtica dioica* L. (vs)	ortica	her	wh	none	Varia/GS	hen	Int (oral)	25		od	93	1723	203
		ortica piccola	her	wh	none	Varia/GS	goat	Int (oral)	26,6		od	89	1730	204
		ortica	her	wh	none	QS	hen	Int (oral)	na		na	na	1803	214
		ortica	her	wh	none	Varia/GS	hen	Int (oral)	4		rd	76	1820	217
		ortica	her	wh	none	QG	hen	Int (oral)	1		rd	98	1854	223
		ortica	her	wh	none	Varia/GS	hen	Int (oral)	1		rd	98	1856	224
		ortica	her	wh	none	Varia/GS	cattle, calf	exin (epicutan)		da	rd	na	1856	224
	Urtica urens L.	ortica	her	wh	none	Varia/GS	goat	Int (oral)	26,6		od	86	1729	204
		ortica(per le galline	lea	wh	none	Varia/GS	hen	Int (oral)	1		ew	99	1749	209
		ortica	her	wh	none	QA	cattle	Int (oral)	0,04		rd	70	1767	211
		ortica	her	wh	none	QA	horse	Int (oral)	0,04		rd	70	1767	211
		ortica	her	wh	none	QA	horse	Int (oral)	0,04		rd	70	1767	211
		ortica	her	wh	none	QA	calf	Int (oral)	0,20		rd	70	1767	211
		ortica	her	wh	none	QA	goat	Int (oral)	0,27		rd	70	1767	211
		ortica	her	wh	none	QA	young goat	Int (oral)	0,53		rd	70	1767	211
		ortica	her	wh	none	QA	hen	Int (oral)	5		rd	67	1767	211
		ortica	her	wh	none	QA	hen	Int (oral)	5		rd	70	1767	211
		ortica	her	wh	water (inf)	QA	cattle	Int (oral)	na		na	na	1784	213
		ortica	her	wh	none	QG	hen	Int (oral)	1		rd	99	1853	223
		ortica	her	wh	none	Varia/GS	hen	Int (oral)	1		rd	99	1855	224
		ortica	her	wh	none	Varia/GS	cattle	exin (epicutan)		da	rd	na	1855	224
		ortica per i maiali	her	wh	water (dec)	Varia/GS	pig	Int (oral)	0,75		ew	90	1878	209

b) German speaking Cantons (Recipe number starts with 1138; Dialog partner starts with 133)

Botanical family	Plant species	Recipe name designated by DP	Plant part	Origin	Extraction procedure on farm	ATCvet Code [21]	Animal treated	Administration	Daily dosage [g/kg0.75]	Conc. [g/100g]	Ver	VAS	RN	DP
**Aloeaceae**	*Aloe vera* (L.) Burm.f.	Aloe Vera	lea	bo*	none	QD	cattle, calf	exal (konj)		na	na	99	1154	136
**Apiaceae**	*Carum carvi* L.	Kümmel	fsb	bo	water (inf)	QA	calf	int (oral)	0,21		od	57	1641	191
	Foeniculum vulgare Mill.	Fencheltee mit Yoghurt	fsb	bo	water (inf)	QA	calf	int (oral)	0,16		od	77	1175	136
		Fencheltee	fsb	bo	water (inf)	QA	calf	int (oral)	0,39		od	74	1666	195
**Araliaceae**	*Panax ginseng* C.A. Meyer	Ginseng	rob	cu	none	GS	calf	int (oral)	0,06		od	91	1665	195
		Ginseng	rob	cu	none	QA	cattle	int (oral)	na		na		1665	195
		Ginseng	rob	cu	water (dec)	GS	bee	int (oral)	na		na		1670	195
		Ginseng	rob	cu	water (dec)	GS	hen	int (oral)	na		na		1670	195
**Aspidiaceae**	*Dryopteris filix-mas* L. (Schott.)	Farn	lea	wh	none	para	hen	tohe		tohe	tohe	77	1682	198
		Farn	her	wh	none	QA	hen	int (oral)	na		na	70	1690	199
**Asteraceae**	*Achillea millefolium* L.	Schafgarbe	her	cu	none	QA	sheep	int (oral)	na		na	71	1144	134
		Schafgarbentee	flo	wh	water (dec)	QA	calf	int (oral)	1,47		ew	80	1151	135
		Schafgarbentee	flo	wh	water (dec)	QA	calf	int (oral)	1,47		ew	92	1151	135
		Schafgarbentee	her	wh	water (inf)	QA	bee	int (oral)	na		na	65	1180	139
		Schafgarbentee	her	wh	water (inf)	behav	cattle	int (oral)	0,39		od	65	1185	139
	Arnica montana L.	Arnikaöl	flo	bo*	oil/fat (-)	mast	cattle	exin (epicutan)		na	na	97	1556	142
		Arnikatinktur	flo	wh	alcohol (rt)	QM	goat	int (oral)	na	0,22	od	94	1574	181
		Arnikatinktur	flo	wh	alcohol (rt)	QD	goat	int (oral)	na	0,22	od	92	1574	181
		Arnikatinktur	flo	wh	alcohol (rt)	QM	goat	exin (epicutan)		0,22	od	90	1574	181
		Arnikatinktur	flo	wh	alcohol (rt)	QM	goat	exin (epicutan)		0,22	od	74	1574	181
		Arnikaöl	flo	bo*	oil/fat (-)	mast	cattle	exin (epicutan)		na	na	78	1600	185
		Arnikaöl	flo	bo*	oil/fat (-)	QG	cattle	exin (epicutan)		na	na	50	1600	185
		Arnikaöl	flo	bo*	oil/fat (-)	QD	cattle	exal (epicutan)		na	na	93	1635	191
		Arnikatinktur	flo	wh	alcohol (rt)	QD	cattle, calf	exal (epicutan)		na	na	59	1701	201
	Artemisia absinthium L.	Wermut-Tee	lea	wh	water (inf)	QA	cattle	int (oral)	0,01		od	75	1200	141
	Calendula officinalis L.	Ringelblumensalbe	flo	cu	oil/fat (rt)	QD	cattle, calf	exal (epicutan)		0,82	rd	95	1148	134
		Ringelblumensalbe	flo	cu	oil/fat (rt)	QD	cattle, calf	exal (epicutan)		0,82	rd	60	1148	134
		Ringelblumensalbe	flo	cu	oil/fat (rt)	QD	hen	exal (epicutan)		0,82	rd	90	1148	134
		Calendula-Tinktur	flo	bo*	n.a.	QD	calf	exal (epicutan)		na	na	97	1192	140
		Ringelblumensalbe	flo	cu	oil/fat (et)	QD	cattle	exal (epicutan)		0,44	rd	70	1558	142
		Ringelblumensalbe	flo	cu	oil/fat (rt)	QD	goat	exal (epicutan)		na	na	93	1567	181

Botanical family	Plant species	Recipe name designated by DP	Plant part	Origin	Extraction procedure on farm	ATCvet Code [21]	Animal treated	Administration	Daily dosage [g/kg0.75]	Conc. [g/100g]	Ver	VAS	RN	DP
**Asteraceae**	*Calendula officinalis* L.	Ringelblumensalbe	flo	cu	oil/fat (et)	mast	cattle	exin (epicutan)		3,33	rd	52	1612	188
		Ringelblumensalbe	flo	cu	oil/fat (et)	QD	cattle	exal (epicutan)		3,33	rd	98	1612	188
		Ringelblumensalbe	flo	cu	oil/fat (et)	QD	cattle, calf	exal (epicutan)		3,33	rd	92	1612	188
		Ringelblumensalbe	flo	cu	oil/fat (et)	QD	cattle	exal (epicutan)		0,84	ew	100	1640	191
		Ringelblumensalbe	flo	cu	oil/fat (et)	QD	cattle	exal (epicutan)		0,84	ew	99	1640	191
		Ringelblumensalbe	flo	cu	oil/fat (et)	QD	horse	exal (epicutan)		0,84	ew	71	1640	191
		Ringelblumensalbe	flo	cu	oil/fat (rt)	QD	cattle	exal (epicutan)		0,96	rd	93	1700	201
		Ringelblumensalbe	flo	cu	oil/fat (rt)	QD	cattle, calf	exal (epicutan)		0,96	rd	97	1700	201
		Ringelblumensalbe	flo	cu	oil/fat (rt)	QD	horse	exal (epicutan)		0,96	rd	83	1700	201
		Ringelblumensalbe	flo	cu	oil/fat (et)	QD	cattle, calf	exin (epicutan)		0,22	rd	85	1587	299
	Matricaria recutita L. (vs*)	Kamillentee	her	wh	water (inf)	GS	cattle	int (oral)	0,13		ew	99	1149	135
		Kamillentee	flo	wh	water (dec)	QA	calf	int (oral)	0,6		rd	87	1150	135
		Kamillentee	flo	bo	water (inf)	QA	calf	int (oral)	0,08		rd	34	1207	142
		Kamillentee	flo	bo	water (inf)	QA	calf	int (oral)	0,29		od	79	1579	182
		Kamillentee	flo	cu	water (dec)	QA	calf	int (oral)	0,08		rd	70	1590	184
		Kamillentee	flo	bo	water (inf)	QA	calf	int (oral)	0,55		rd	97	1597	185
		Kamillentinktur	flo	wh	alcohol (rt)	QD	cattle, calf	exal (epicutan)		3,52	rd	77	1605	187
		Kamillentinktur	flo	wh	alcohol (rt)	QD	cattle, calf	exal (epicutan)		3,52	rd	87	1605	187
		Kamillentee	flo	wh	water (dec)	QA	calf	int (oral)	0,07		ew	88	1606	187
		Kamillentee	flo	cu	water (inf)	QA	pig	int (oral)	0,28		rd	68	1615	188
		Kamillentee	flo	cu	water (inf)	varia	pig	int (oral)	0,06		rd	57	1615	188
		Kamillentee	flo	cu	water (inf)	QA	calf	int (oral)	0,09		rd	70	1615	188
		Kamillentee	flo	cu	water (inf)	QG	cattle	exal (epicutan)		0,15	rd	47	1615	188
		Kamillentee	flo	cu	water (inf)	QG	pig	exin (epicutan)		0,15	rd	63	1615	188
		Kamillentee	flo	bo	water (inf)	QA	calf	int (oral)	0,16		rd	77	1618	189
		Kamillentee	flo	bo	water (inf)	QA	cattle	int (oral)	0,05		rd	100	1597	191
		Kamillentinktur	flo	bo*	n.a.	QD	cattle, calf	exal (epicutan)		na	na	54	1636	191
		Kamillentinktur	flo	bo*	n.a.	QD	horse	exal (epicutan)		na	na	57	1636	191
		Kamillentee	her	cu	water (inf)	QA	calf	int (oral)	2,47		od	100	1639	191
		Kamillentee	her	cu	water (inf)	QA	calf	int (oral)	1,88		od	100	1639	191
		Kamillentee	flo	bo	water (inf)	QA	calf	int (oral)	0,34		od	72	1650	193
		Kamillentee	flo	wh	water (inf)	QA	calf	int (oral)	0,53		rd	55	1672	196
		Kamillentee	flo	wh	water (inf)	QD	calf	exal (epicutan)		0,6	rd	53	1672	196
		Kamillentee	flo	wh	water (inf)	QD	calf	exal (epicutan)		0,6	rd	65	1672	196

Botanical family	Plant species	Recipe name designated by DP	Plant part	Origin	Extraction procedure on farm	ATCvet Code [21]	Animal treated	Administration	Daily dosage [g/kg0.75]	Conc. [g/100g]	Ver	VAS	RN	DP
**Asteraceae**	*Matricaria recutita* L.(vs*)	Kamillentee	flo	bo	water (inf)	QA	calf	int (oral)	0,71		od	82	1683	198
		Kamillentee	flo	bo	water (inf)	QA	cattle	int (oral)	0,12		od	34	1683	198
		Kamillentee	flo	bo	water (inf)	QA	calf	int (oral)	0,53		od	88	1683	198
		Kamillentee	her	wh	water (inf)	QA	calf	int (oral)	0,78		od	56	1688	199
		Kamillentee	flo	bo	water (inf)	QD	cattle	exal (konj)		0,5	od	50	1699	201
		Kamillentee	flo	bo	water (inf)	QD	hen	exal (epicutan)		0,5	od	80	1699	201
		Kamillentee	flo	bo	water (inf)	QD	horse	exal (konj)		0,5	od	na	1699	201
		Kamillentee	flo	wh	water (inf)	QA	cattle	int (oral)	0,03		rd	na	1708	202
		Kamillentee	flo	wh	water (inf)	QD	cattle, calf	exal (epicutan)		0,22	rd	na	1708	202
**Berberidaceae**	*Berberis vulgaris* L.	Berberitzenäste	twb	wh	none	QD	cattle, calf	tohe		tohe	tohe	71	1174	136
		Berberitze	twb	wh	none	QD	cattle, calf	tohe		tohe	tohe	100	1681	197
		Berberitze	twb	wh	none	QD	calf	tohe		tohe	tohe	79	1689	199
**Boraginaceae**	*Symphytum officinale* L. (vs*)	Beinwellblätter	lea	cu	none	QA	calf	int (oral)	0,5		ew	47	1146	134
		Beinwellblätter	lea	cu	none	QA	cattle	int (oral)	0,1		ew	47	1146	134
		Beinwellsalbe	rob	cu	oil/fat (et)	QD	cattle, calf	exal (epicutan)		0,35	od	86	1147	134
		Beinwellsalbe	rob	cu	oil/fat (et)	QM	cattle, calf	exin (epicutan)		0,35	od	81	1147	134
		Wallwurzsalbe	rob	bo*	alcohol (-)	mast	cattle	exin (epicutan)		na	na	84	1557	142
		Wallwurzsalbe	rob	bo*	alcohol (-)	QM	cattle	exin (epicutan)		na	na	69	1557	142
		Beinwellsalbe	rob	wh	oil/fat (et)	QD	pig	exal (epicutan)		na	na	88	1584	183
		Beinwellsalbe	rob	wh	alcohol (rt)	QD	pig	exal (epicutan)		na	na	62	1584	183
		Beinwellsalbe	rob	wh	oil/fat (et)	QM	pig	exin (epicutan)		na	na	87	1584	183
		Beinwellsalbe	rob	wh	alcohol (rt)	QM	pig	exin (epicutan)		na	na	87	1584	183
		Beinwellsalbe	rob	wh	oil/fat (et)	QM	pig	exin (epicutan)		na	na	87	1584	183
		Beinwellsalbe	rob	wh	alcohol (rt)	QM	pig	exin (epicutan)		na	na	87	1584	183
		Wallwurzsalbe	rob	bo*	n.a.	mast	cattle	exin (epicutan)		na	na	98	1599	185
		Wallwurzsalbe	rob	wh	water (dec)	QM	cattle, calf	exin (epicutan)		0,05	rd	79	1608	187
		Wallwurzsalbe	rob	wh	water (dec)	QM	cattle, calf	exin (epicutan)		0,05	rd	78	1608	187
		Wallwurzgel	rob	bo*	n.a.	mast	cattle	exin (epicutan)		na	na	94	1622	189
		Wallwurzgel	rob	bo*	alcohol (-)	QD	cattle, calf	exal (epicutan)		na	na	99	1634	191
**Brassicaceae**	*Armoracia rusticana* G.Gaertn., B.Mey. & Scherb.	Meerrettich	rob	cu	none	QA	pig	int (oral)	0,28		od	94	1654	194
		Meerrettichtee	rob	cu	water (inf)	QA	calf	int (oral)	1,18		od	82	1655	194
		Meerrettich	rob	cu	none	GS	cattle	int (oral)	3,89		od	81	1656	194
		Meerrettich	rob	cu	none	QM	cattle, calf	exin (epicutan)		1,33	od	89	1657	194

Botanical family	Plant species	Recipe name designated by DP	Plant part	Origin	Extraction procedure on farm	ATCvet Code [21]	Animal treated	Administration	Daily dosage [g/kg0.75]	Conc. [g/100g]	Ver	VAS	RN	DP
**Brassicaceae**	*Armoracia rusticana* G.Gaertn., B.Mey. & Scherb.	Meerrettich	rob	cu	none	mast	cattle	exin (epicutan)		1,33	od	57	1657	194
**Brassicaceae**	*Capsella bursa-pastoris* L.	Hirtentäschli	her	wh	none	QG	cattle	int (oral)	na		na	100	1199	141
**Cannabaceae**	*Cannabis sativa* L.	Hanfsalbe	flo	bo*	n.a.	mast	cattle	exin (epicutan)		na	na	84	1592	184
		Hanfsalbe	her	cu	oil/fat (et)	mast	cattle	exin (epicutan)		na	na	70	1671	196
		Hanfsalbe	her	cu	oil/fat (et)	varia	cattle	exin (epicutan)		na	na	75	1671	196
		Hanfsalbe	her	cu	oil/fat (et)	varia	cattle	exin (epicutan)		na	na	83	1671	196
		Hanfsalbe	her	cu	oil/fat (et)	QM	cattle	exin (epicutan)		na	na	88	1671	196
**Caprifoliaceae**	*Sambucus nigra* L.	Holder	twb	wh	none	QA	cattle	na		da	na	na	1716	202
**Ericaceae**	*Arctostaphylos uva-ursi* (L.) Spreng.	Bärentraubenblätter tee	lea	bo	water (dec)	QG	cattle	int (oral)	0,13		od	91	1193	140
**Fagaceae**	*Quercus robur* L.	Eichenrindentee	bar	bo	water (inf)	QA	pig	int (oral)	1,69		rd	69	1138	133
		Eichenrinde	bar	bo	none	QA	calf	int (oral)	0,72		rd	82	1209	142
		Eichenrindentee	bar	bo	water (inf)	QA	calf	int (oral)	0,6		rd	49	1210	142
		Eichenrinde	bar	bo	none	QA	calf	int (oral)	1,33		rd	81	1617	189
		Eichenrindentee	bar	bo	water (inf)	QA	calf	int (oral)	2,82		rd	72	1629	190
		Eichenrindenpulver	bar	bo	none	QA	calf	int (oral)	0,94		ew	67	1630	190
		Eichenrinde	bar	bo	none	QA	pig	int (oral)	0,008		rd	18	1596	191
**Hypericaceae**	*Hypericum perforatum* L.(vs)	Johannisöl	flo	cu	oil/fat (rt)	QG	cattle	exin (epicutan)		0,3	ew	86	1143	134
		Johanniskrautöl	flo	wh	oil/fat (rt)	QM	goat	exin (epicutan)		na	na	93	1569	181
		Johanniskrautöl	her	wh	oil/fat (rt)	QD	cattle	exal (epicutan)		na	na	79	1702	201
**Juglandaceae**	*Juglans regia* L.	Nussbaumspray	lea	wh	water (dec)	para	goat	exin (epicutan)		na	na	94	1570	181
**Lamiaceae**	*Origanum vulgare* L.	Oreganoöl	n.a	bo*	oil/fat (-)	QA	hen	int (oral)	na		na	81	1677	197
		Oreganoöl	n.a	bo*	oil/fat (-)	QA	hen	int (oral)	na		na	93	1677	197
	Thymus vulgaris L.	Thymian	her	bo	none	QA	calf	int (oral)	0,14		rd	99	1196	140
		Thymian	her	bo	none	QR	calf	int (oral)	0,78		rd	96	1196	140
		Thymian	her	bo	none	varia	calf	int (oral)	0,14		rd	97	1196	140
		Thymian	her	bo	none	varia	cattle	int (oral)	0,31		rd	99	1196	140
		Thymian	her	bo	none	mast	cattle	int (oral)	0,07		rd	78	1213	142
		Thymiantee	her	bo	water (inf)	QA	calf	int (oral)	0,34		rd	56	1598	185
		Thymiantee	her	bo	water (inf)	QD	pig	exal (epicutan)		0,43	rd	83	1598	185
		Thymiantee	her	bo	water (inf)	QA	calf	int (oral)	0,98		rd	82	1621	189
		Thymiantee	her	bo	water (inf)	QR	calf	int (oral)	0,19		rd	43	1621	189
		Thymian	her	bo	none	QR	calf	int (oral)	0,16		od	99	1637	191
		Thymiantee	her	bo	water (inf)	QR	calf	int (oral)	0,37		od	83	1638	191

Botanical family	Plant species	Recipe name designated by DP	Plant part	Origin	Extraction procedure on farm	ATCvet Code [21]	Animal treated	Administration	Daily dosage [g/kg0.75]	Conc. [g/100g]	Ver	VAS	RN	DP
**Lauraceae**	*Cinnamomum camphora* L.	Kampfersalbe	n.a	bo*	none	mast	cattle	exin (epicutan)		na	na	46	1183	139
	Laurus nobilis L.	Lorbeerfrüchte-Versäuberungspulver	fsb	bo*	water (inf)	QG	cattle	int (oral)	na		na	52	1197	140
**Liliaceae**	*Allium cepa* L.	Zwiebel	rob	cu	water (rt)	GS	cattle	int (oral)	0,33		rd	68	1604	187
		Zwiebel	rob	cu	water (rt)	QA	cattle	int (oral)	0,33		rd	64	1604	187
**Linaceae**	*Linum usitatissimum* L.	Flachschleim	fsb	bo	water (inf)	QA	calf	int (oral)	1,51		rd	82	1588	184
		Leinsamen	fsb	bo	none	QG	cattle	int (oral)	0,28		rd	na	1703	201
		Leinsamen	fsb	bo	water (inf)	QG	cattle	int (oral)	na		na	na	1714	202
**Malvaceae**	*Althaea officinalis* L.	Eibischsalbe	rob	cu	water (dec)	mast	cattle	exin (epicutan)		na	na	61	1182	139
		Eibischsalbe	rob	bo	oil/fat (rt)	mast	cattle	exin (epicutan)		na	na	52	1611	188
	Malva sylvestris L.	Käslikraut	her	wh	water (inf)	QD	cattle, calf	exal (epicutan)		na	na	na	1709	202
**Papaveraceae**	*Chelidonium majus* L.	Schöllkraut	lea	cu	none	QD	cattle, calf	exal (epicutan)		da	na	55	1589	184
**Pinaceae**	*Abies alba* Mill.	Weisstannenäste	twb	wh	none	QR	calf	int (nasal)		na	na	96	1177	136
		Weisstannenharz	exc	wh	none	QD	cattle, calf	exal (epicutan)		da	na	56	1642	192
	Picea abies (L.)H. Karst.	Harzsalbe	exc	wh	oil/fat (et)	QD	goat	exal (epicutan)		9,92	ew	97	1568	181
		Harzsalbe	exc	wh	oil/fat (et)	QD	goat	exal (epicutan)		9,92	ew	97	1568	181
		Harzsalbe	exc	wh	oil/fat (et)	QD	goat	exal (epicutan)		9,92	ew	96	1568	181
		Tannenäste	twb	wh	none	QR	calf	int (oral)	na		na	53	1620	189
**Poaceae**	*Avena sativa* L.	Habersuppe	fsb	bo	water (inf)	QA	calf	int (oral)	19,61		ew	93	1208	142
		Haferschleim	fsb	bo	water (inf)	QA	calf	int (oral)	20,59		od	74	1667	195
**Polygonaceae**	*Rumex obtusifolius* L. (vs*)	Blackentee	rob	wh	water (dec)	QA	calf	int (oral)	0,8		od	86	1188	139
		Blackentee	rob	wh	water (dec)	QA	calf	int (oral)	0,16		na	87	1189	139
		Blackenöl	lea	wh	oil/fat (rt)	QD	cattle, calf	exal (epicutan)		23,72	od	100	1201	141
		Blackenöl	lea	wh	oil/fat (rt)	QD	cattle, calf	exal (epicutan)		23,72	od	100	1201	141
		Blackentinktur	lea	wh	alcohol (rt)	QD	cattle, calf	exal (epicutan)		0,01	rd	97	1204	141
		Blackentinktur	lea	wh	alcohol (rt)	QD	cattle, calf	exal (epicutan)		0,01	rd	99	1204	141
		Blackentinktur	lea	wh	alcohol (rt)	QM	cattle, calf	exal (epicutan)		0,01	rd	100	1204	141
		Blackentinktur	lea	wh	alcohol (rt)	QM	cattle, calf	int (oral)	na		na	98	1204	141
		Blacke	wpr	wh	none	QA	pig	int (oral)	na		na	53	1562	180
		Blackentee	rob	wh	water (dec)	QA	calf	int (oral)	1,57		od	78	1575	182
**Rhamnaceae**	*Rhamnus catharticus* L.	Kreuzdorn	twb	wh	none	QD	cattle, calf	tohe		tohe	tohe	74	1142	134
		Kreuzdorn	twb	cu	none	QD	cattle, calf	tohe		tohe	tohe	51	1181	139
		Kreuzdorn	twb	cu	none	QD	cattle, calf	tohe		tohe	tohe	81	1563	180

Botanical family	Plant species	Recipe name designated by DP	Plant part	Origin	Extraction procedure on farm	ATCvet Code [21]	Animal treated	Administration	Daily dosage [g/kg0.75]	Conc. [g/100g]	Ver	VAS	RN	DP
**Rhamnaceae**	Rhamnus catharticus L.	Kreuzdorn	twb	wh	none	QD	calf	tohe		tohe	tohe	65	1591	184
		Kreuzdorn	twb	wh	none	QD	cattle, calf	tohe		tohe	tohe	77	1601	186
		Kreuzdorn	twb	wh	none	QD	cattle, calf	tohe		tohe	tohe	91	1660	195
		Kreuzdorn	twb	wh	none	QD	calf	tohe		tohe	tohe	62	1675	196
		Kreuzdorn	twb	wh	none	QD	calf	tohe		tohe	tohe	58	1687	198
**Rosaceae**	Alchemilla mollis (Buser) Rothm.	Frauenmäntelitee	lea	wh	water (inf)	QD	cattle	exal (epicutan)		0,03	rd	84	1178	136
		Frauenmäntelitee	lea	wh	water (inf)	QD	cattle, calf	exal (epicutan)		0,03	rd	77	1178	136
		Frauenmänteli	her	cu	none	QG	cattle	int (oral)	0,31		od	64	1211	142
	Malus domestica Borkh.	Zimtapfel	fsb	bo	none	QG	cattle	int (oral)	na		na	27	1212	142
	Potentilla anserina L.	Gänsefingerkraut	lea	wh	water (dec)	QA	calf	int (oral)	0,02		rd	45	1593	184
	Potentilla erecta (L.) Räuschel (vs*)	Blutwurztinktur	rob	wh	alcohol (rt)	QD	cattle, calf	exal (epicutan)		na	na	91	1583	183
		Blutwurztinktur	rob	wh	alcohol (rt)	QD	cattle, calf	exal (epicutan)		na	na	92	1583	183
		Blutwurztinktur	rob	wh	alcohol (rt)	QD	pig	exal (epicutan)		na	na	88	1583	183
		Tormentillpulver	rob	bo	water (rt)	QA	calf	int (oral)	2,16		od	100	1695	200
		Tormentillpulver	rob	bo	milk (rt)	QA	calf	int (oral)	1,73		od	na	1697	200
**Rubiaceae**	Coffea spp.	Kafi-Schnaps	fsb	bo	water (inf)	QA	cattle	int (oral)	0,05		ew	83	1141	134
		Kafi-Schnaps	fsb	bo	water (inf)	QA	cattle	int (oral)	0,16		rd	59	1565	180
		Kafi-Schnaps	fsb	bo	water (inf)	varia	cattle	int (oral)	0,16		rd	82	1565	180
		Kafi-Schnaps	fsb	bo	water (inf)	GS	cattle	int (oral)	0,16		rd	79	1602	187
		Kafi-Schnaps	fsb	bo	water (inf)	QA	cattle	int (oral)	0,07		rd	73	1651	193
		Kafi-Schnaps	fsb	bo	water (inf)	QA	cattle	int (oral)	0,07		rd	62	1651	193
		Kafi-Schnaps	fsb	bo	water (inf)	GS	cattle	int (oral)	0,23		rd	86	1658	194
		Kaffeebohnensatz	fsb	bo	water (inf)	QA	calf	int (oral)	9,8		od	76	1668	195
		Kafi-Schnaps	fsb	bo	water (inf)	GS	cattle	int (oral)	na		na	68	1673	196
		Kafi-Schnaps	fsb	bo	water (inf)	QA	cattle	int (oral)	na		na	78	1678	197
		Kafi-Schnaps	fsb	bo	water (inf)	QA	cattle	int (oral)	0,04		rd	74	1693	199
**Rutaceae**	*Citrus x limon* (L.) Burm.f.	Zitronenessig	fsb	bo	water (inf)	GS	cattle	int (oral)	0,78		rd	100	1205	142
		Zitronenessig	fsb	bo	water (inf)	GS	cattle	int (oral)	0,78		rd	97	1205	142
**Scrophulariaceae**	*Euphrasia rostkoviana* Hayne	Augentrostsalbe	her	wh	oil/fat (rt)	QD	goat	exal (konj)		na	na	91	1572	181
		Augentrostöl	her	wh	oil/fat (rt)	QD	goat	exal (konj)		na	na	95	1573	181
**Theaceae**	*Camellia sinensis* (L.) O. Kuntze	Schwarztee	lea	bo	water (inf)	QA	calf	int (oral)	0,27		od	80	1578	182
		Schwarztee	lea	bo	water (inf)	QA	calf	int (oral)	0,39		od	66	1647	193
		Schwarztee	lea	bo	water (inf)	QA	calf	int (oral)	0,39		od	75	1648	193

Botanical family	Plant species	Recipe name designated by DP	Plant part	Origin	Extraction procedure on farm	ATCvet Code [21]	Animal treated	Administration	Daily dosage [g/kg0.75]	Conc. [g/100g]	Ver	VAS	RN	DP
**Theaceae**	*Camellia sinensis* (L.) O. Kuntze.	Schwarztee	lea	bo	water (inf)	QA	calf	int (oral)	0,47		od	88	1704	201
		Schwarztee	lea	bo	water (inf)	QA	calf	int (oral)	0,37		rd	na	1710	202
**Urticaceae**	*Urtica dioica* L. (vs*)	Brennessel	her	cu	none	QA	hen	int (oral)	0,6		ew	73	1145	134
		Brennesselblätter	lea	bo	none	varia	cattle	int (oral)	0,16		na	47	1194	140
		Brennesselblätter	lea	bo	none	QA	cattle	int (oral)	0,05		rd	77	1560	142
		Brennesselkraut	her	wh	none	QA	goat	int (oral)	na		na	93	1571	181
		Brennesselkraut	her	wh	none	GS	calf	int (oral)	na		na	88	1580	182
		Brennesselkraut	her	wh	none	QA	calf	int (oral)	na		na	63	1580	182
		Brennesseltee	lea	bo	water (inf)	varia	calf	int (oral)	0,08		rd	69	1619	189
		Brennesseltee	lea	bo	water (inf)	varia	calf	int (oral)	0,08		rd		1619	189
		Brennessel	lea	wh	none	behav	pig	int (oral)	na		na	74	1631	190
		Brennessel	her	wh	none	QA	cattle	int (oral)	na		na	93	1684	198
		Brennessel	her	wh	none	QA	pig	int (oral)	na		na	93	1684	198
