# Supplementary material for: Swiss ethnoveterinary knowledge on medicinal plants – a within-country comparison of Italian speaking regions with north-western German speaking regions
Source: J Ethnobiol Ethnomed. 2017 Jan 3;13:1. doi: 10.1186/s13002-016-0106-y (PMC5209851; doi:10.1186/s13002-016-0106-y)
Supplement: Additional file 2: — List of all reported plant species used to treat animals in West Alps regions. (PDF 139 kb) [file 13002_2016_106_MOESM2_ESM.pdf]

Additional Document II – List of all reported plant species used to treat animals in West Alps regions

Abbreviation:

Own data
ItR: Italian speaking Region of our study
GeC: German speaking Cantons of our study

Reference data
CH1: [23]: Schmid K, Ivemeyer S, Vogl C, Klarer F, Meier B, Hamburger M, Walkenhorst M: Traditional use of herbal remedies in livestock by farmers in 3 swiss cantons (aargau, zurich, schaffhausen). Forschende Komplementarmedizin 2012;19:125-136.
CH2: [22]: Disler M, Ivemeyer S, Hamburger M, Vogl C, Tesic A, Klarer F, Meier B, Walkenhorst M: Ethnoveterinary herbal remedies used by farmers in four north-eastern swiss cantons (st. Gallen, thurgau, appenzell innerrhoden and appenzell ausserrhoden). Journal of Ethnobiology and Ethnomedicine 2014;10:32.
IT1: [24]: Pieroni A, Giusti ME: Alpine ethnobotany in italy: Traditional knowledge of gastronomic and medicinal plants among the occitans of the upper varaita valley, piedmont. Journal of Ethnobiology and Ethnomedicine 2009;5
IT2: [26]: Vitalini S, Iriti M, Puricelli C, Ciuchi D, Segale A, Fico G: Traditional knowledge on medicinal and food plants used in val san giacomo (sondrio, italy) - an alpine ethnobotanical study. Journal of Ethnopharmacology 2013;145:517-529.
IT3: [27]: Vitalini S, Tomè F, Fico G: Traditional uses of medicinal plants in Valvestino (italy). Journal of Ethnopharmacology 2009;121:106-116.
IT4: [28]: Mattalia G, Quave CL, Pieroni A: 74 Traditional uses of wild food and medicinal plants among Brigasc, Kyé, and Provençal communities on the Western Italian Alps. Genetic Resources and Crop Evolution 2013;60:587-603.
IT5: [25]: Cornara L, La Rocca A, Marsili S, Mariotti MG: Traditional uses of plants in the eastern riviera (Liguria, Italy). Journal of Ethnopharmacology 2009;125:16-30.

Plant species	IsR	GsC	CH	Italian data
Abies alba **Mill.**	x	x	CH2	
Achillea millefolium **L.**	x	x	CH2	IT1, IT2, IT3
**Achillea moschata Jacq.**				IT2
Alchemilla alpina **L. or** ***Alchemilla conjuncta*** **Bab.**				IT2
Alchemilla mollis **(Buser) Rothm.**		x		
Alchemilla vulgaris **L. em. S.E. Frohner or** ***Alchemilla xanthochlora*** **Rothm.**			CH2	IT2
Aleopecurus pratensis **L.**				IT4
Allium cepa **L.**	x	x	CH1, CH2	
Allium sativum **L.**	x		CH2	
Aloe vera **(L.) Burm.f.**		x	CH1	
Allium ursinum **L.**	x			
Althaea officinalis **L.**		x	CH2	
Anthyllis vulneraria **L.s.str.**			CH2	
Arctostaphylos uva-ursi **(L.) Spreng.**		x		
Armoracia rusticana **G.Gaertn., B.Mey. & Scherb.**		x		
Arnica chamissonis **Less.**			CH2	

Arnica montana **L.**	x	x	CH1, CH2	IT2
Artemisia absinthium **L.**	x	x	CH1	IT4, IT1, IT2
Artemisia campestris **L.**	x			
Artemisia vulgaris **L.**				IT1
Arum Italicum **Mill.**				IT5
Avena sativa **L.**	x	x	CH1, CH2	IT1
Beta vulgaris **subsp.** ***vulgaris*** **(*conditiva*- group)**			CH2	
Betula pendula **Roth.**			CH2	
Berberis vulgaris **L.**		x		
Brassica napus **L.**			CH1, CH2	
Brassica oleracea **L.**	x		CH2	IT1
Calendula officinalis **L.**	x	x	CH1, CH2	
Camellia sinensis **(L.) O. Kuntze.**		x	CH1, CH2	
Cannabis sativa **L.**		x	CH2	
Capsella bursa- pastoris **L.**		x	CH2	
Carum carvi **L.**		x	CH1, CH2	
Castanea sativa **Mill.**	x			
Cetraria islandica **(L.) Ach.**				IT2
Chelidonium majus L.		x	CH1	
Chenopodium bonus-henricus **L.**			CH2	IT1
Cinnamomum camphora **L.**		x		
Cinnamomum verum **J.Presl.**	x		CH2	
Citrus x limon **(L.) Burm.f.**		x	CH2	
Clematis vitalba **L.**				IT5
Coffea **L.**	x	x	CH1, CH2	
Crataegus laevigata **(Poir.) DC. or** ***Crataegus monogyna Jacq.***			CH2	
Cucurbita maxima **Duch.**	x			
Dryopteris filix- mas **(L.) Schot.**	x	x	CH2	
Equisetum arvense **L.**			CH2	IT5
Equisetum ramosissimum **Desf.**				IT5
**Equisetum telemateia Ehrh.**				IT5
**Eugenia caryophillata Thunb.**	x			
**Eucalyptus globulus Labill.**			CH1	
Euphrasia rostkoviana **Hayne**	x	x	CH1, CH2	
Festuca arundinaceae **Schreb.**				IT4
Ficus carica **L.**				IT5
Filipendula ulmaria **(L.) Maxim.**			CH1	
Foeniculum vulgare **Mill.**	x	x	CH1, CH2	IT5
Fraxinus excelsior **L.**			CH2	
Fregaria vesca **L.**				IT4
Gentiana acaulis **L.**				IT4
Gentiana lutea **L.**			CH2	IT4
Gentiana punctata **L.**				IT2
Gentiana purpurea **L.**	x			IT2
Gentiana verna **L.**				IT4
Geranium robertianum **L.**			CH1, CH2	

Helianthus annuus **L.**			CH2	
Helleborus foetidus **L.**				IT5
Helleborus viridis **L.**				IT5
Hordeum vulgare **L.**	x			
Humulus lupulus **L.**			CH1	
Hyoseris radiate **L.**				IT5
Hypericum perforatum **L.**	x	x	CH1, CH2	IT5
Ilex aquifolium **L.**			CH2	
Juglans regia **L.**	x	x	CH1, CH2	
Juniperi communis **ssp. Alpina Celak.**			CH2	
Juniperus communis **L.**				IT5
Juniperus oxycedrus **L.**				IT5
Laburnum alpinum **(Mill.) Berecht. & J.Presl**				IT1
Larix decidua **Mill.**				IT2
Laurus nobilis **L.**		x		
Lavandula angustifolia **Mill.**	x		CH1, CH2	
Linum usitatissimum **L.**	x	x	CH1, CH2	
Lycopodium clavatum **L.**			CH2	
Malus domestica **Borkh.**		x	CH2	
Malva neglecta **Wallr.**	x		CH1, CH2	IT2, IT3
Malva sylvestris **L.**	x	x	CH1	
Matricaria recutita **L.**	x	x	CH1, CH2	IT5
Melaleuca alternifolia **(Maiden&Betche) Cheel.**			CH2	
Mentha arvensis **var piperascens**			CH1	
Mentha **x** ***piperita*** **L.**			CH2	
Mercurialis annua **L.**				IT5
Myristica fragrans **Houtt.**			CH1, CH2	
Nicotiana tabacum **L.**	x			
Olea europaea **L.**	x		CH2	
Oreganum majorana **L.**			CH1	
Origanum vulgare **L.**		x	CH1	
Oryza sativa **L.**	x			
Panax ginseng **C.A. Meyer**		x		
Parietaria officinalis **L.**				IT5
Pedicularis verticillata **L.**			CH2	
Pelargonium sidoides **DC**			CH2	
Petroselinum crispum **(Mill.) Fuss**			CH2	
Peucedanum ostruthium **(L.) W.D.J.Koch**				IT1
Picea abies **(L.) H. Karst.**	x	x	CH1, CH2	IT2
Plantago lanceolata **L.**			CH1, CH2	IT5, IT1
Plantago major **L. s.l.**				IT5, IT1, IT2
Potentilla anserina **L.**		x		
Potentilla erecta **(L.) Räuschel.**	x	x	CH2	
Prunus domestica **L.**			CH2	
Prunus spinosa **L.**			CH2	
Quassia amara L.			CH2	

Quercus robur L. or *Quercus petraea* (Matt.) Liebl.	x	x	CH1, CH2	
Ranunculus bulbosus L.				IT5
Reichardia picroides (L.) Roth.				IT5
Rhamnus catharticus L.	x	x	CH1, CH2	
Rhododendron ferrugineum L.				IT2
Ricinus communis L.	x			
Rubus idaeus L.			CH2	
Rumex alpinus L.				IT1, IT2
Rumex crispus L.				IT5
Rumex obtusifolius L.		x	CH1, CH2	IT5
Rumex sanguineus L.				IT5
Ruta angustifolia Pers.				IT5
Ruta chalapensis L.				IT5
Ruta graveolens L.				IT5
Salix caprea L.			CH2	
Salvia officinalis L.			CH1, CH2	
Salvia verbenacea L.	x			
Sambucus nigra L.		x	CH2	
Sanicula europaea L.			CH1, CH2	
Senecio alpinus (L.)			CH2	
Senecio ovatus (G. Gaertn. & Al.) Willd.			CH2	
Solanum tuberosum L.			CH1, CH2	
Solidago virgaurea L.			CH2	
Sonchus oleraceus L.				IT5
Stellaria media (L.) Vill.	x			
Symphytum officinale L.	x	x	CH1, CH2	
Tanacetum parthenium (L.) Sch. Bip.			CH2	
Tanacetum vulgare L.			CH2	
Taraxacum officinale WEB. Ex Wigg.	x		CH1	
Thuja occidentalis L.			CH1, CH2	
Thymus pulegioides L.				IT5
Thymus vulgaris L.		x	CH1, CH2	IT5
Tilia cordata Mill. or *Tilia platyphyllos* Scop.			CH1, CH2	
Triticum aestivum L.	x			
Tropaeolum majus L.			CH1, CH2	
Urtica dioica L.	x	x	CH1, CH2	IT5
Urtica urens L.	x			
Vaccinium myrtillus L.			CH2	
Veratrum album L.				IT4, IT1, IT2, IT3
Veronica allionii Vill.				IT1
Viscum album L.	x			
Vitis vinifera L.			CH1	
